# Antifungal activity of isolated compounds from C*entaurea salicifolia* var. *salicifolia* against *Candida* species, including multidrug-resistant *C. auris*, *C. inconspicua*, and *C. dubliniensis*

**DOI:** 10.55730/1300-0527.3906

**Published:** 2026-03-17

**Authors:** Selin TUFAN, Dilek ŞATANA, Nur TAN

**Affiliations:** 1 Department of Pharmacognosy, Faculty of Pharmacy, İstanbul University, İstanbul, Turkiye; 2 Department of Medical Microbiology, İstanbul Medical Faculty, İstanbul University, İstanbul, Turkiye

**Keywords:** Antifungal, *Candida*, *Candida auris*, flavonoids, natural compounds, sesquiterpene lactones

## Abstract

Natural compounds obtained from plants have always held great potential for drug discovery and, as such, have drawn the attention of researchers as novel antifungal molecules that can be effective against fungal resistance. The antifungal activity of sesquiterpene lactones has been extensively investigated against various fungal strains, but there is limited data on their antifungal activity against *Candida* species. Sesquiterpene lactones are abundant in the Asteraceae family, and 90% of identified sesquiterpene lactones have been isolated from members of this family. Three extracts obtained from the aerial parts of *Centaurea salicifolia* var. *salicifolia*, which had not been investigated prior to this study, were tested for their antifungal activity against *Candida albicans* (ATCC 90028), *C. dubliniensis* (NCPF 3949a), *C. auris* (NCPF 8977), *C. inconspicua* (NCPF 8523), and *C. krusei* (ATCC 6258) and exhibited minimum inhibitory concentration (MIC) values ranging between 6.25 μg/mL and 12.5 μg/mL. Due to its rich sesquiterpene lactone content, isolation studies conducted on a dichloromethane maceration extract (CsDCM-M) yielded cynaropicrin (SL1), vahlenin (SL2), maximolide (SL3), pectolinaringenin (F1), centaureidin (F2), and axillarin (F3).

The isolation of vahlenin from plants has been reported in only two studies, published in 1974 and 1978; thus, the present work constitutes the first documented isolation of vahlenin since the 1970s. Relative stereochemistry elucidation along with the 2D-NMR (COSY, HSQC, HMBC, and NOESY) data for vahlenin is given for the first time. Antifungal tests against the abovementioned *Candida* strains were also performed using all of the isolated compounds, and they exhibited promising MIC values between 12.5 μg/mL and 50 μg/mL. This is the first report on the antifungal activity of vahlenin and maximolide against *C. auris*, *C. inconspicua*, and *C. dubliniensis*.

## 1. Introduction

The emerge of fungal resistance has hastened research on new antifungal lead compounds against multidrug-resistant *Candida* strains such as *Candida inconspicua*, *C. dubliniensis*, and *C. auris.* The Centers for Disease Control and Prevention declared *C. auris* as ‘urgent’ because of its resistance against all three available antifungal groups [1–3]. Apart from the rising antifungal resistance, the safety, efficacy and cost limitations of the current antifungal agents restricts their use in treatment. Their toxicity requires caution during treatment, and adverse effects caused by the current antifungal agents are not well-tolerated by patients. Also, drug interactions may increase their toxicity or decrease their efficacy. The efficacy of an antifungal agent does not solely depend on its potency against the pathogen but also relies on its ability to reach and maintain sufficient concentration at the infection site. The pharmacokinetic properties of the current antifungal agents, such as low oral bioavailability, high protein binding, and tissue penetration problems, can also hinder antifungal treatment. All of these limitations of the current antifungal agents result in greater hospitalization, unsuccessful antifungal treatment, and increased economic burden on the healthcare system [4,5].

Natural compounds originating from plants have always drawn attention for their antibacterial, antiviral, and antifungal properties and have been employed for research into novel antifungal molecules. The structural diversity of natural compounds, especially flavonoids and sesquiterpene lactones (SLs), provides a great source for discovery of novel molecules against the arising antifungal resistance. Their diversity facilitates the targeting of different mechanisms for eluding antifungal resistance. Flavonoids exhibit antifungal activity by targeting the fungal cell wall with mechanisms such as binding ergosterol and modulating oxidative pathways, and studies have demonstrated [6,7]that flavonoids in combination with the current antifungal agents may increase their antifungal efficacy [6,7]. On the other hand, SLs exert antifungal activity by targeting fungal membranes. The literature states that their lipophilic structure enables interference with mitochondrial function, modulation of oxidative pathways, and disruption of membrane integrity. Both flavonoids and SLs display antifungal activity via various mechanisms, and they can be used alone or in combination with the current antifungal agents against multidrug-resistant strains. Moreover, the differing pharmacokinetic properties of the natural compounds compared to the current antifungal agents may increase their efficacy [8,9].

The Asteraceae family is a great source for sesquiterpene lactones (SLs) and flavonoids. SLs have always been noted for their antifungal properties, and 90% of identified SLs have been isolated from the Asteraceae family. Besides SLs, flavonoids have also been employed in antifungal research including in vivo tests [8,10], and the extracts and natural compounds obtained from the *Centaurea* L. genus have been used in antifungal research [11].

Many previous studies have been done in this area of research. In one study, the antifungal activity of nine extracts obtained from the leaves, flowers, and roots of *C. tchihatcheffii* were assessed against *C. albicans* and *C. parapsilosis*. The extracts gave minimum inhibitory concentration (MIC) values between 4 μg/mL and 8 μg/mL, which was determined to be significant compared to fluconazole and ketoconazole [12]. In another study, extracts obtained from 10 *Centaurea* sp. were tested against *C. glabrata* and *C. krusei.* Six of the ten *Centaurea* sp. collected from Türkiye exhibited antifungal activity against *C. krusei* (MIC = 15–100 μg/mL). Especially, the MIC values of *C. depressa* and *C. urvillei* subsp. *urvillei* hexane extracts (MIC values of 15 and 45 μg/mL, respectively) were significant compared to amphotericin B (MIC = 2 μg/mL) [13]. The antifungal activity of five Turkish endemic *Centaurea* sp., namely *C. amanicola, C. kurdica, C. odyssei, C. ptosimopappa*, and *C. ptossimopappoides*, were tested against *C. albicans* and *C. glabrata.* Ethanol, acetone, ethyl acetate, and chloroform extracts were obtained via a Soxhlet apparatus. The extracts gave a 8–24 mm inhibition zone against *C. albicans* and *C. glabrata* at a concentration of 100 mg/mL (ketoconazole, 17 and 21 mm, respectively) [14]. Sequential hexane, ethyl acetate, and ethanol Soxhlet extracts from Turkish endemic *Centaurea cariensis* subsp*. niveo-tomentosa* were tested against *C. albicans* and *C. tropicalis*. None of the extracts exhibited antifungal activity against *C. albicans* while the chloroform and ethyl acetate extracts showed 14 mm and 24 mm inhibition zones against *C. tropicalis*, respectively [15]. Isolation studies on *Centaurea zuccariniana* yielded three new SLs that showed higher antifungal activity than bifonazole and ketoconazole against *C. albicans*, with zuccarinin being the most potent among the tested SLs [16]. Chlorohyssopifolin A, chlorojanerin, and 13-acetylsolstitialin A isolated from Turkish *Centaurea solstitialis* subsp. *solstitialis* also exhibited antifungal activity against *C. albicans* and *C. parapsilosis* with 64 μg/mL MIC values [17]. Besides the SLs, there are several in vitro and in vivo reports on antifungal activity of flavonoids against *Candida* strains as well. The combination of flavonoids such as quercetin and isoquercetin with fluconazole increased its efficacy against *C. albicans* [18,19].

Evaluation of these studies revealed that the *Centaurea* L. taxa and its natural compounds present a great potential to overcome antifungal resistance, especially against multidrug-resistant *Candida* strains. The aim of this study is to investigate the antifungal potential of extracts and natural compounds obtained from *Centaurea salicifolia* var. s*alicifolia*, which has not been previously researched. All of the isolated compounds were subjected to antifungal test against *Candida albicans* (ATCC 90028), *C. krusei* (ATCC 6258), *C. inconspicua* (NCPF 8523), *C. dubliniensis* (NCPF 3949a), and *C. auris* (NCPF 8977).

## 2. Material and methods

### 2.1. Plant material and extraction

*Centaurea salicifolia* var. *salicifolia* was collected from Şavşat, Artvin, Türkiye in August 2018 and identified by Prof. Dr. Emine Akalın. The sample is stored at the herbarium of the Faculty of Pharmacy at İstanbul University (ISTE) with voucher number ISTE 117052. The plant material was coarsely chopped and dried in a moisture-free room away from direct light.

The coarsely chopped dry plant material was macerated twice at room temperature for 20 min with dichloromethane (coded as CsDCM-M). After maceration, the plant material was dried, powdered, and extracted with dichloromethane (coded as CsDCM-S) and methanol (coded as CsMeOH-S) (Merck, Germany) sequentially in a Soxhlet apparatus. All of the extracts were dried under vacuum at 45 °C with a rotary evaporator (Buchi, Switzerland).

### 2.2. Isolation studies

The CsDCM-M extract (4570 mg) was fractionated in a Sephadex LH-20 (25–100 μm, Sigma Chemical Co., Germany) column and DCM:MeOH (3:1, v/v) was used as the mobile phase. TLC (TLC silica gel 60 F_254_, aluminum) examination of fractions showed that Fr.61–81 yielded the pure sesquiterpene lactone SL1 (cynaropicrin, 1800 mg). SL1 was the major compound in the extract, but Fr. 38–55 (106 mg) was also subjected to Sephadex LH-20 column with hexane: dichloromethane: methanol (7:4:1 v/v/v) as the mobile phase, which gave subfraction Fr. 36–54 (20.3 mg). Preparative silica gel TLC (PLC silica gel 60 F_254_, 1 mm, glass, Sigma Chemical Co., Germany) of Fr. 36–54 yielded SL2 (vahlenin, 2.3 mg), SL3 (maximolide, 3 mg), and flavonoid F1 (pectolinaringenin, 2.2 mg). Fractionation of Fr. 82–89 (129 mg) and Fr. 102–104 (27.2 mg) in a Sephadex LH-20 column with methanol yielded F2 (centaureidin, 8.2 mg) and F3 (axillarin, 7.4 mg), respectively. All of the solvents were purchased from Merck, Germany.

### 2.3. Structure elucidation

Nuclear Magnetic Resonance (NMR) was employed for structure elucidation of the isolated compounds. The NMR data were recorded on a Bruker Ascend NEO NMR Spectrometer (USA) operating at 500 and 125 MHz for ^1^H and ^13^C, respectively. The IR of SL2 (vahlenin) was determined using a Bruker Alpha FT-IR (USA). The LC-MS analysis of SL2 was performed with a Thermo Scientific Q Exactive spectrometer (USA), and optical rotation was measured in methanol using a Rudolph Analytical Autopol V Plus (USA). The UV spectra of vahlenin were recorded using a UV-Vis Spectrophotometer (UV-1700, Shimadzu, Japan). Spectral data of all of the compounds are given in the supplementary data.

The spectroscopic data of SL2 (vahlenin); white amorphous powder; [α]^23.2^_D_: +50° (*c* 0.2 mg/mL, MeOH); UV (*c*, 0.05 mg/mL) (MeOH) λ_max_ (log ɛ) nm: 212 (3.89), 250 (2.51), 330 (2.39); ^1^H and ^13^C APT (Table 1); (+)-HRESIMS: *m/z* 373.1617 [M + Na]^+^ (calcd *m/z* 373.1622); IR V_max_ (NaCl) cm^−1^: 3432, 2919, 2851, 1759, 1709, 1625, 1588, 1166, 1131, 1018.

### 2.4. Antifungal activity

The antifungal activity of the extracts and isolated compounds was tested against *Candida albicans* ATCC 90028 (American Type Culture Collection, USA), *C. dubliniensis* NCPF 3949a (National Collection of Pathogenic Fungi, UK Health Security Agency), *C. auris* NCPF 8977, *C. inconspicua* NCPF, and *C. krusei* ATCC 6258. The microdilution method was employed according to the standard protocol of the Clinical and Laboratory Standard Institute (CLSI). Amphotericin B (Sigma-Aldrich, Germany), the extracts, and the isolated compounds were dissolved in 100% dimethyl sulfoxide as recommended in the CLSI guidelines [20–22]. The final concentrations of all extracts and isolated compounds were 50–0.002 μg/mL, and amphotericin B was 16–0.007 μg/mL.

Resazurin (Sigma-Aldrich, St. Louis, USA) was dissolved in sterile distilled water to a final concentration of 0.02%, sterilized by filtration, and stored at 4 °C until use [23]. A sterility check of each bottle was performed before use. The broth microdilution test was performed using sterile, disposable microdilution plates (96 U-shaped wells) (LP Italiano SPA, Milan, Italy). RPMI 1640 broth with L-glutamine without sodium bicarbonate and 0.165 M MOPS buffer (34.54 g/L) were used. The medium was adjusted to pH 7.0 at 25 °C.

Preparation of the inoculum suspensions of yeasts was largely based on the CLSI guidelines [20–22]. After incubation at 35 °C for 48 h on Sabouraud dextrose agar (BBL, Sparks, MD, USA), the yeast colonies were subcultured into 5 mL of sterile saline (0.85%). The turbidity was adjusted spectrophotometrically at 530 nm to a 0.5 McFarland standard, followed by two dilutions (1:50, then 1:20) in RPMI 1640 to obtain a final concentration of 0.5 × 10^3^ to 2.5 × 10^3^ CFU/mL.

Serial dilution of the samples and amphotericin B was done with RPMI 1640 medium and each row contained serial dilutions of the samples in 100 μL media (<2% DMSO/RPMI). In the third microplate, row G contained serial dilutions of amphotericin B, rows H1–H3 contained the DMSO control (< 2% DMSO/RPMI), rows H4–H6 contained a positive control (no sample), and rows H7–H12 contained a negative control (broth only). Each well was inoculated on the day of the test with 100 μL of the corresponding inoculum, except the negative control. This step brought the drug dilutions and inoculum size to the final test concentrations given above. The microplates were incubated at 35 °C for 24–48 h. The minimum concentration at which no growth was observed was taken as the MIC value [20–23]. The MIC values were also confirmed using resazurin [23]. The resazurin solution was added to each well (30 μL), and the plates were incubated for one more day. A purple color was defined as negative, and a pink color was defined as positive for fungal growth. The first purple-colored well was accepted as the MIC value [20,23].

## 3. Results and discussion

### 3.1. Isolation studies and structure elucidation

**Figure 1:**
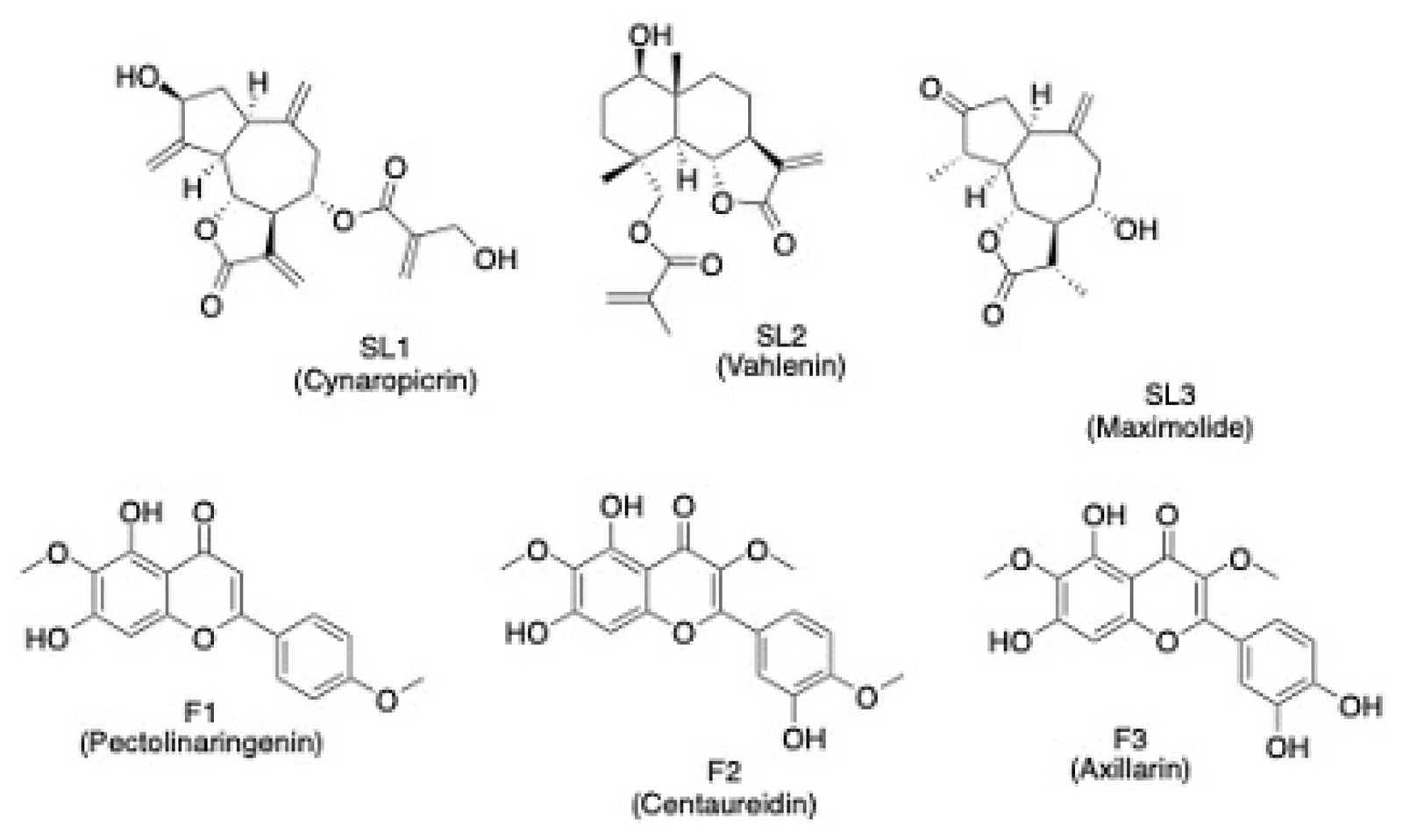
Chemical structures of the isolated compounds.

The structures of the compounds isolated from *C. salicifolia* var. *salicifolia* are given in Figure 1. SL1 was the major compound in the CsDCM-M extract. According to the ^1^H NMR spectra taken in CDCl_3_, SL1 was identified as cynaropicrin [24], which was previously isolated from *Cynara scolymus* [25] and from various *Centaurea* species such as *C. solstitialis* [26], *C. behen* [27], *C. helenioides* [28], and Turkish endemic *C. drabifolia* subsp. *detonsa*. SL2 was determined to be vahlenin, previously isolated from *C. hyssopifolia* [29] and *C. linifolia* [30] in the 1970s, and having no other reported isolations until now. The ^1^H and ^13^C NMR spectra of SL2 are compatible with the literature, but 2D-NMR experiments (^1^H-^1^H COSY, HSQC, HMBC, and NOESY) were not conducted on vahlenin. The ^1^H-^1^H COSY, HMBC, and NOE correlations are given in Table 1. Furthermore, the relative stereochemistry elucidation of vahlenin is given for the first time. The NMR data of SL3 is in agreement with the literature on maximolide [31], which was isolated from taxa of the Asteraceae family, *Ainsliaea spicata* [32], *Hemisteptia lyrata* [33], and *Amberboa lippi* [34], which is also known as *Centaurea lippi* [35]. F1 (pectolinaringenin) [36], F2 (centaureidin), and F3 (axillarin) [37] were also previously isolated from various *Centaurea* taxa [38–40]. All of the spectral data of the isolated compounds, including the 2D-NMR spectra of vahlenin, are given in the supplementary data.

### 3.2. 2D-NMR data and relative stereochemistry elucidation of vahlenin

**Figure 2:**
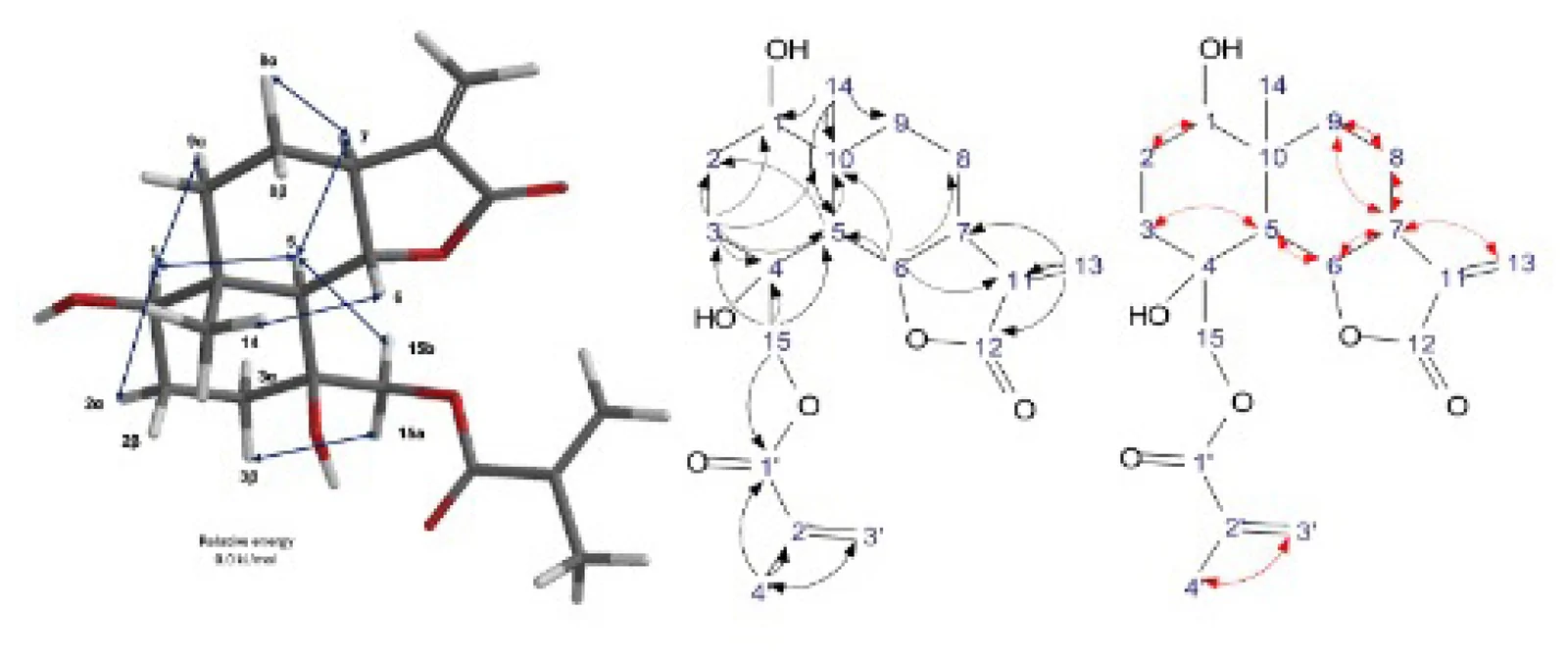
The ^1^H-^1^H NOE, ^1^H-^1^H COSY, and HMBC correlations of SL2 (vahlenin).

Despite the prior identification of vahlenin, its 2D-NMR data are presented herein for the first time (Table 2). The HMBC correlation of δ_c_ 170.1 (C-12) is a characteristic signal of the lactone moiety, with H_2_-13 (δ_H_ 6.09 and 5.42) signals substantiating the presence of *α*-methylene γ-lactone. The HMBC correlation between δ_H_ 6.09 (H-13a) and quaternary δ_c_ 139.0 (C-11), in addition to the prominent ^1^H-^1^H COSY correlation between H_2_-13 protons and the H-7 proton (δ_H_ 2.51), reinforce the structure assignment. ^1^H-^1^H COSY correlations among H-1, H-2, H-5, H-6, H-7, H-8, and H-9 corroborate the proposed structure (Figure 2). The methyl signal H_3_-14 (δ_H_ 1.18) exhibits distinct HMBC correlations with C-1 (δ_c_ 78.6), C-5 (δ_c_ 52.3), C-9 (δ_c_ 38.0), and C-10 (δ_c_ 42.5), establishing its position. The quaternary δ_c_ 72.9 (C-4) signal shows HMBC correlations with the H-3*α* (δ_H_ 1.80), H-5 (δ_H_ 1.56), and H_2_-15 AB signals (δ_H_ 4.77 and 4.08). In addition to C-4, the H_2_-15 signals also showed HMBC correlation with an acid carbonyl signal at δc 168.1 (C-1′). The ^1^H-^1^H COSY correlation between H_3_-4′ (δ_H_ 1.95) and H_2_-3′ (δ_H_ 5.60 and 6.11) protons, along with the prominent HMBC correlations of H_3_-4′ with C-2′ (δ_c_ 136,1), C-3′ (δc 126,3), and C-1′ (δc 168,1) supported methacrylic acid conjugation.

The ^1^H-^1^H NOESY spectrum displayed no correlation between H-6 and H-7, which indicates *trans*-fusion of the lactone ring to a six-membered ring. The H-7↔H-5 and H-5↔H-1 NOE correlations marked position of those protons to *α*. Likewise, the H-6↔H-14 NOE correlation determined the 14-CH_3_ place to be *β*. Also, the absence of correlation between H-6 and H-15 showed that C4-OH’s place is *β*. All of the NOE correlations are given in Figure 2.

### 3.3. Antifungal activity

Fungal infections caused by *Candida* sp., especially *C. albicans*, have always posed a threat to human health. There are antifungal agent groups used against *Candida* strains, but the rise of the fungal resistance has led to invasive fungal infections [41,42]. Along with that, the emergence of other multidrug-resistant *Candida* strains such as *C. inconspicua*, *C. dubliniensis*, and *C. auris* has expedited research on novel antifungal compounds. This need turned the attention of research groups to natural compounds such as SLs and flavonoids due to their antimicrobial potential [1–3,10].

The antifungal activity of SLs has been widely investigated, but their mechanism of action is not completely understood. The literature suggests that SLs interfere with membrane integrity due to their lipophilicity and alkylation of cellular enzymes [43]. Various reports on the antifungal activity of SLs favor the presence of an *α*-methylene γ-lactone ring due to its alkylation capacity function [44–46]. Unfortunately, the *α*-methylene γ-lactone ring is also responsible for the toxic effects of SLs [10,16,47–51]. The antifungal activity of flavonoids is also well documented. Previous studies have demonstrated that flavonoids exhibit antifungal activity by targeting fungal cell membranes and biofilm formation [6, 52].

The antifungal activity of natural compounds against *C. albicans* has been widely explored. Flavonoids have been investigated for their antifungal activity against *Candida* strains. Notably, there are limited data on the antifungal activity of SLs against *Candida* strains considering the comprehensive antifungal studies on other fungal strains. Among the tested SLs, only SL1 (cynaropicrin) was tested against *C. albicans* prior to this study. Schinor et al. assessed the antifungal activity of cynaropicrin using the agar disk diffusion method and reported a 500 μg/mL MIC value against *C. albicans* (ATCC 1023), whereas in this study, cynaropicrin gave a MIC value of 12.5 μg/mL against *C. albicans* (ATCC 90028) [53]. This pronounced difference between MIC values of cynaropicrin may be attributed to the use of different *C. albicans* strains, which can vary in susceptibility. Additionally, the CLSI broth microdilution method employed in the present study could also account for the difference [20–22]. In a previous study, our research group reported the antifungal activity of SLs, including cynaropicrin, isolated from *C. polypodiifolia* against the same *Candida* strains used in the present study. As expected, cynaropicrin from the aforementioned study exhibited identical MIC values since the same strains and methodology were employed [54].

Three different extracts obtained from *Centaurea salicifolia* var. *salicifolia* were investigated for their antifungal activity against *C. albicans*, *C. krusei*, *C. inconspicua*, *C. dubliniensis*, and *C. auris*. The antifungal activity of the extracts and isolated compounds against these strains is given in Table 2.

The antifungal activities of SLs and flavonoids on *C. krusei* have been investigated previously [7,55]. The current results show that the CsDCM-M, CsDCM-S and CsMeOH-S extracts exhibited the best antifungal activity against *C. krusei* among the tested strains with 6.25 μg/mL MIC values. All of the extracts had the same MIC (12.5 μg/mL) value against the other tested strains. Isolation studies were conducted on the CsDCM-M extract due to its rich SL content.

All of the isolated compounds had the same MIC value as the CsDCM-M extract (12.5 μg/mL) against *C. albicans*, multidrug-resistant *C. dubliniensis*, and *C. inconspicua*. Unlike its antifungal activity against *C. albicans* and *C. dubliniensis*, SL1 (cynaropicrin) exhibited two-fold lower activity (MIC of 25 μg/mL) than the remaining isolated compounds against *C. auris*. For *C. krusei*, the CsDCM-M extract showed higher antifungal activity (MIC = 6.25 μg/mL) than the isolated compounds. SL2 (vahlenin) displayed four-fold higher antifungal activity (MIC = 12.5 μg/mL) while the MIC values of the other tested compounds stayed at 50 μg/mL against *C. krusei*.

All of the isolated compounds exhibited equal or lower antifungal activity against the other tested strains than the CsDCM-M extract. This might be attributed to synergistic effects caused by the simultaneous targeting of different pathways as demonstrated by previous studies [6]. Tenoria et al. investigated the antifungal activity of spray-dried crude extract from *Eugenia uniflora* leaves, the ethyl acetate fraction of the crude extract, and enriched subfractions against *C. albicans* (ATCC 90028), *C. glabrata* (ATCC 9001), and *C. auris* (CDC B11903). Subfractions enriched individually with myricitrin, gallic acid, and ellagic acid had similar or higher MIC values than the crude extract. Only the ellagic acid enriched subfraction exhibited two-fold higher antifungal activity than the crude extract and ethyl acetate fraction against *C. glabrata* (MIC of 62.5 μg/mL). Also, myricitrin, gallic acid, ellagic acid, and their combinations displayed higher MIC values than their enriched fractions and crude extract against all of the tested strains. Especially against *C. auris*, pure compounds and their combinations showed MIC values ranging between 500–1000 μg/mL, while crude extract had a MIC value of 31.2 μg/mL. It was suggested that natural compounds may act in conjunction with the extract [56]^.^

SL1 (cynaropicrin) and SL3 (maximolide) had the same MIC values against all of the tested strains except *C. auris*. The antifungal mechanism of SLs is still not fully understood, but the literature suggests it could be based on disruption of fungal membranes due to their lipophilicity and the alkylation of cellular enzymes. Also, the lipophilic character of SLs appears advantageous in terms of penetration through the fungal wall [43]. Previous reports on the antifungal activity of SLs favor the presence of an *α*-methylene γ-lactone ring due to its alkylation capacity, which does not correlate with our results. Özçelik et al. [17] isolated centaurepensin, chlorojanerin, and 13-acetylsolstitialin A from *Centaurea solstitialis* ssp. *solstitialis* and investigated their antifungal activity against *C. albicans* and *C. parapsilosis*. The only SL that does not bear *α*-methylene γ-lactone among the other two SLs is 13-acetylsolstitialin, but it still had the same MIC value (64 μg/mL) against the tested two strains. Eremanthin, a guaianolide with an *α*-methylene γ-lactone ring, did not exhibit antifungal activity at 250 μg/mL against *C. albicans* (MTC 227) [57]. The antifungal activities of eight distinct guaianolides against the same *Candida* species used in the present study have been evaluated, highlighting the potential of guaianolides without an *α*-methylene-*γ*-lactone ring as promising antifungal agents by our research group [54]. These findings substantiate the need for exploration of antifungal activity of SLs without an *α*-methylene γ-lactone ring. Besides the lactone ring variations, all of the guaianolides mentioned above bear structural differences that can affect their antifungal activity. Two eudesmanolides isolated from *Centaurea zuccariniana*, sonchucarpolide derivative and zuccarinin, displayed higher antifungal activity than bifonazole and ketoconazole against *C. albicans* [16]. The eudesmanolide SL2 (vahlenin) showed equal or higher antifungal activity against all of the tested strains with SL1 and SL3 guaianolides. Especially against *C. krusei*, SL2 (vahlenin) showed a four-fold higher antifungal activity (MIC = 12.5 μg/mL) than all other isolated compounds. This might be explained by the more lipophilic nature of eudesmanolides than guaianolides.

Even though these results and the literature suggest that the presence of an *α*-methylene γ-lactone ring is not the only factor in the antifungal activity of SLs, further studies are still needed for complete understanding of the SAR (structure activity relationship) in antifungal activity. The isolation of structurally diverse SLs from natural sources and semisynthesis studies will enable better understanding of the antifungal SAR. Further antifungal activity mechanism studies are still needed in order to explore guaianolides as antifungal molecules against *Candida* strains. Along with their antifungal properties, the cytotoxicity of SLs must also be assessed to evaluate their safety.

## 4. Conclusion

The emergence of fungal resistance and the limitations of current antifungal agents has accelerated research into novel antifungal agents, especially from natural compounds due to their structural diversity and antifungal properties. This study investigates the natural compounds and antifungal activity of extracts obtained from *Centaurea salicifolia* var. *salicifolia* for the first time. Isolation studies conducted on the dichloromethane maceration extract yielded three SLs and three flavonoids. As vahlenin has not been isolated since the 1970s, its relative stereochemistry had never been established. Thus, in the present study, the 2D-NMR data of vahlenin and its relative stereochemistry according to NOESY 2D-NMR spectra is given for the first time.

All of the tested natural compounds showed promising antifungal activity against the tested strains. Previous studies on the antifungal activity of SLs focused on polarity and alkylation capacity. Unfortunately, alkylation capacity, linked to the presence of an *α*-methylene γ-lactone ring, is also related to the toxicity of SLs, which may limit their usefulness. The antifungal activity results of cynaropicrin and maximolide against all of the tested strains revealed that SLs with an *α*-methyl γ-lactone ring might show equal antifungal activity to SLs with an *α*-methylene γ-lactone ring. Further studies on the mechanism and SAR of SLs are needed to better understand their antifungal activity. Isolation of SLs with different functional groups and semisynthesis studies are required to expand the research on antifungal activity against *Candida* strains.

## Supplementary Information



## Figures and Tables

**Table 1:** ^1^H and ^13^C (APT) chemical shifts and HMBC, COSY, NOESY correlations of SL2 (vahlenin).

No	δ _H_	δ _C_	HMBC	COSY	NOESY
1	3.36 (m, 1H)	78.6	-	H2b, H2a	H5, H2a, H9b
2a	1.67 (m, 1H)	26.4	-	H1, H2b	H1
2b	1.91 (m, 1H)		-	H1, H2a	H14
3*α*	1.80 (ddd, *J* = 14.4, 4.1, 2.7 Hz, 1H)	34.1	C1, C5, C4	H5, H3*β*	-
3*β*	1.56 (m, 1H) ^*2^		C10, C2	H3*α*	H15a
4	-	72.9	H3*α*, H15a	-	-
5	1.56 (m, 1H) ^*2^	52.3	C10, C2	H3*α*, H6	H1, H7, H15b
6	4.29 (t, *J* = 10.9 Hz, 1H)	79.5	C10, C5, C8b, C11	H5, H7	H14
7	2.51 (m, 1H)	50.9	-	H9a, H8b, H8a, H6, H13b, H13a	H5, H9b, H8a
8a	2.05 (m, 1H) ^*1^	21.6	-	H9b, H8b, H7	H9b, H7
8b	1.67 (m, 1H)		-	H9b, H9a, H8a, H7	-
9a	2.05 (m, 1H)^*1^	38.0	-	H9b, H8b, H7	H9b
9b	1.29 (dd, *J* = 13.5, 4.3 Hz, 1H)		-	H9a, H8b, H8a	H1, H9a, H8a, H7
10	-	42.5	H5, H3*β*, H6, H14	-	-
11	-	139.0	H6, H13b, H13a	-	-
12	-	170.1	H13b, H13a	-	-
13a	6.09 (d, *J* = 3.2 Hz, 1H)	117.4	C7, C11, C12	H7	H13b
13b	5.42 (d, *J* = 3.0 Hz, 1H)		C7, C11, C12	H7	H13a
14	1.18 (d, *J* = 0.8 Hz, 3H)	-	C1, C10, C5, C9b	-	H2b, H6
15a	4.77 (d, *J* = 11.8 Hz, 1H)	73.6	C5, C4, C3, C1′	H15b	H3*β*, H15b
15b	4.08 (d, *J* = 11.8 Hz, 1H)		C5, C3, C1′	H15a	H5, H15a
1′	-	168.1	H15b, H15a, H4′	-	-
2′	-	136.1	H4′	-	-
3′a	5.60 (p, *J* = 1.5 Hz, 1H)	126.3	C4′	H4′, H3′b	H4′, H3′b
3′b	6.11 (q, *J* = 1.2 Hz, 1H)		C4′	H4′, H3′a	H3′a
4′	1.95 (t, *J* = 1.3 Hz, 3H)	18.5	C1′, C2′, C3′b	H3′b, H3′a	H3′a

*1 and *2 = overlapping signals.

**Table 2:** MIC values of extracts and natural compounds (μg/mL).

	MIC range: 50–0.002 μg/mL (12 dilutions)				
	*Candida albicans* ATCC 90028	*Candida auris* NCPF 8971	*Candida dubliniensis* NCPF 3949a	*Candida krusei* ATCC 6258	*Candida inconspicua* NCPF 8523
CsDCM-M	12.5	12.5	12.5	**6.25**	12.5
CsDCM-S	12.5	12.5	12.5	**6.25**	12.5
CsMeOH-S	12.5	12.5	12.5	**6.25**	12.5
SL1 (Cynaropicrin)	12.5	25	12.5	50	12.5
SL2 (Vahlenin)	12.5	12.5	12.5	12.5	12.5
SL3 (Maximolide)	12.5	12.5	12.5	50	12.5
F1 (Pectolinaringenin)	12.5	12.5	12.5	50	12.5
F2 (Centaureidin)	12.5	12.5	12.5	50	12.5
F3 (Axillarin)	12.5	12.5	12.5	50	12.5
Amphotericin B (16–0.007 μg/mL)	≤ 0.007	0.014	≤0.007	2	≤ 0.007
DMSO control	+	+	+	+	+
Positive control	+	+	+	+	+
Negative control	-	-	-	-	-

CsDCM-M: *Centaurea salicifolia* var. *salicifolia* dichloromethane maceration extract; CsDCM-S: *C. salicifolia* var. *salicifolia* dichloromethane Soxhlet extract;

CsMeOH-S: *C. salicifolia* var. *salicifolia* methanol Soxhlet extract
