# Supplementary material for: Antifungal activity of isolated compounds from Centaurea salicifolia var. salicifolia against Candida species, including multidrug-resistant C. auris, C. inconspicua, and C. dubliniensis
Source: Turk J Chem. 2026 Mar 17;50(4):412–22. doi: 10.55730/1300-0527.3906 (PMC13533064; doi:10.55730/1300-0527.3906)
Supplement: Supplementary file 1 [file tjc-50-04-412suppmaterials.pdf]

# Supporting Information

**Antifungal activity of isolated compounds from *Centaurea salicifolia* var. *salicifolia* against *Candida* species, including multidrug-resistant *C. auris*, *C. inconspicua*, and *C. dubliniensis***


Selin TUFAN[1], Dilek ŞATANA[2], Nur TAN[1,*]

[1]Department of Pharmacognosy, Faculty of Pharmacy, İstanbul University, İstanbul, Türkiye

[2]Department of Medical Microbiology, İstanbul Medical Faculty, İstanbul University, Türkiye

*Correspondence: nurtan@istanbul.edu.tr

ORCIDs:

Selin TUFAN: https://orcid.org/0000-0002-9173-3423

Dilek ŞATANA: https://orcid.org/0000-0002-8827-1504

Nur TAN: https://orcid.org/0000-0001-7958-1917

## List of Figures

Figure S1: SL1 (Cynaropicrin) 1D-$^{1}$H Proton NMR Spectrum (500 MHz, $CDCl_3$) .............................. 4

Figure S2 : SL2 (Vahlenin) $^{1}$H Proton 1D-NMR Spectrum (500 MHz, $CDCl_3$) .................................. 6

Figure S3 : SL2 (Vahlenin) $^{13}$C (APT) 1D-NMR Spectrum (125 MHz, $CDCl_3$) .................................. 7

Figure S4 : SL2 (Vahlenin) COSY 2D-NMR Spectrum .................................................................. 8

Figure S5 : SL2 (Vahlenin) HSQC 2D-NMR Spectrum .................................................................. 9

Figure S6 : SL2 (Vahlenin) HMBC 2D-NMR Spectrum .................................................................. 10

Figure S7 : SL2 (Vahlenin) NOESY 2D-NMR Spectrum ................................................................ 11

Figure S8 : SL2 (Vahlenin) Mass Spectrum (*m/z* 373.1617 $[M + Na]^+$ (calcd *m/z* 373.1622); error: -.499 ppm) .................................................................................................................. 12

Figure S9 : SL2 (Vahlenin) IR Spectrum ....................................................................................... 13

Figure S10 : SL2 (Vahlenin) UV spectrum (in methanol, 0.05 mg/mL) ............................................... 14

Figure S11: SL3 (Maximolide) 1D-$^{1}$H Proton NMR Spectrum (500 MHz, $CDCl_3$) ............................ 16

Figure S12: SL3 (Maximolide) $^{13}$C (APT) 1D-NMR (125 MHz, $CDCl_3$) ........................................... 17

Figure S13: SL3 (Maximolide) COSY 2D-NMR Spectrum ................................................................ 18

Figure S14 : SL3 (Maximolide) HSQC 2D-NMR Spectrum .............................................................. 19

Figure S15: SL3 (Maximolide) HMBC 2D-NMR Spectrum .............................................................. 20

Figure S16 : SL3 (Maximolide) NOESY 2D-NMR Spectrum ........................................................... 21

Figure S17 : F1 (Pectolinaringenin) 1D-$^{1}$H Proton NMR Spectrum (500 MHz, $CDCl_3$) ..................... 22

Figure S18: F1 (Pectolinaringenin) $^{13}$C (APT) 1D-NMR (125 MHz, $CDCl_3$) ..................................... 23

Figure S19 : F2 (Centaureidin) 1D-$^{1}$H Proton NMR Spectrum (500 MHz, $CDCl_3$) ............................ 24

Figure S 20 : F2 (Centaureidin) $^{13}$C (APT) 1D-NMR (125 MHz, $CDCl_3$) ......................................... 25

Figure S21: F3 (Axillarin) 1D-$^{1}$H Proton NMR Spectrum (500 MHz, MeOD) .................................. 26

Figure S22: F3 (Axillarin) $^{13}$C (APT) 1D-NMR (125 MHz, MeOD) ................................................. 27

## List of Tables

Table S1: SL2 (Vahlenin) HMBC, COSY, NOESY corelations ......................................................... 5

Table  S2: SL3 (Maximolide) HMBC, COSY, NOESY corelations .................................................... 12

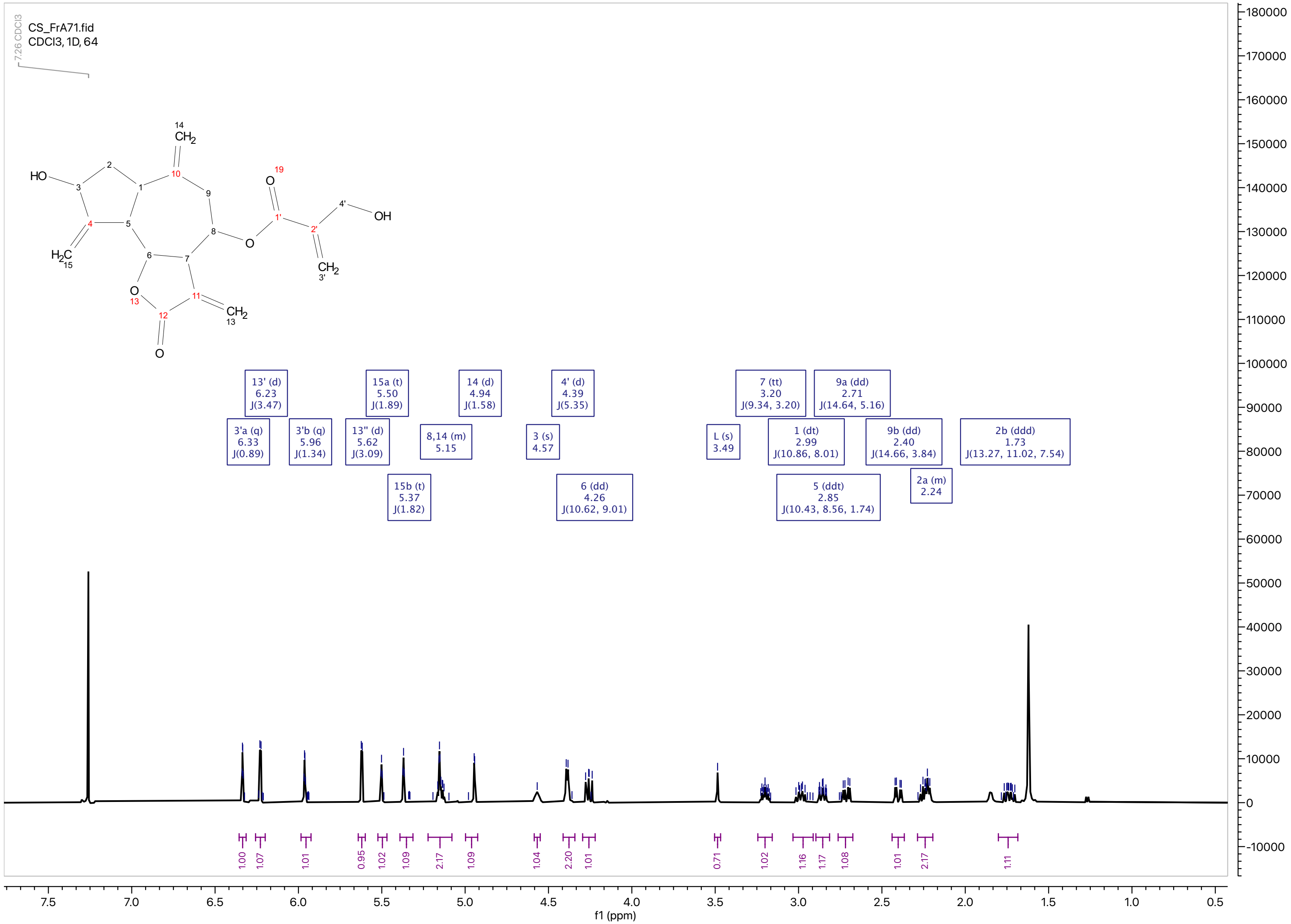


**Figure S1:** SL1 (Cynaropicrin) 1D-$^{1}$H Proton NMR Spectrum (500 MHz, $CDCl_3$)

**Table S1:** SL2 (Vahlenin) HMBC, COSY, NOESY corelations

| No | HMBC | COSY | NOESY |
|---|---|---|---|
| 1 | - | H2b(1.91), H2a(1.67) | H5(1.55), H2a(1.67), H9b(1.29) |
| 2a | - | H1(3.36), H2b(1.91) | H1(3.36) |
| 2b | - | H1(3.36), H2a(1.67) | H14(1.18) |
| 3α | C1(78.6), C5(52.3), C4(72.9) | H5(1.55), H3β(1.56) | - |
| 3β | C10(42.5), C2b(26.4) | H3α(1.80) | H15a(4.77) |
| 4 | H3α(1.80), H15a(4.77) | - | - |
| 5 | C10(42.5), C2b(26.4) | H3α(1.80), H6(4.29) | H1(3.36), H7(2.51), H15b(4.08) |
| 6 | C10(42.5), C5(52.3), C8b(21.6), C11(139.0) | H5(1.55), H7(2.51) | H14(1.18) |
| 7 | - | H9a(2.05), H8b(1.67), H8a(2.05), H6(4.29), H13b(5.42), H13a(6.09) | H5(1.55), H9b(1.29), H8a(2.05) |
| 8a | - | H9b(1.29), H8b(1.67), H7(2.51) | H9b(1.29), H7(2.51) |
| 8b | - | H9b(1.29), H9a(2.05), H8a(2.05), H7(2.51) | - |
| 9a | - | H9b(1.29), H8b(1.67), H7(2.51) | H9b(1.29) |
| 9b | - | H9a(2.05), H8b(1.67), H8a(2.05) | H1(3.36), H9a(2.05), H8a(2.05), H7(2.51) |
| 10 | H5(1.55), H3β(1.56), H6(4.29), H14(1.18) | - | - |
| 11 | H6(4.29), H13b(5.42), H13a(6.09) | - | - |
| 12 | H13b(5.42), H13a(6.09) | - | - |
| 13a | C7(50.9), C11(139.0), C12(170.1) | H7(2.51) | H13b(5.42) |
| 13b | C7(50.9), C11(139.0), C12(170.1) | H7(2.51) | H13a(6.09) |
| 14 | C1(78.6), C10(42.5), C5(52.3), C9b(38.0) | - | H2b(1.91), H6(4.29) |
| 15a | C5(52.3), C4(72.9), C3β(34.1), C1'(168.1) | H15b(4.08) | H3β(1.56), H15b(4.08) |
| 15b | C5(52.3), C3β(34.1), C1'(168.1) | H15a(4.77) | H5(1.55), H15a(4.77) |
| 1' | H15b(4.08), H15a(4.77), H4'(1.95) | - | - |
| 2' | H4'(1.95) | - | - |
| 3'a | C4'(18.5) | H4'(1.95), H3'b(6.11) | H4'(1.95), H3'b(6.11) |
| 3'b | C4'(18.5) | H4'(1.95), H3'a(5.60) | H3'a(5.60) |
| 4' | C1'(168.1), C2'(136.1), C3'b(126.3) | H3'b(6.11), H3'a(5.60) | H3'a(5.60) |

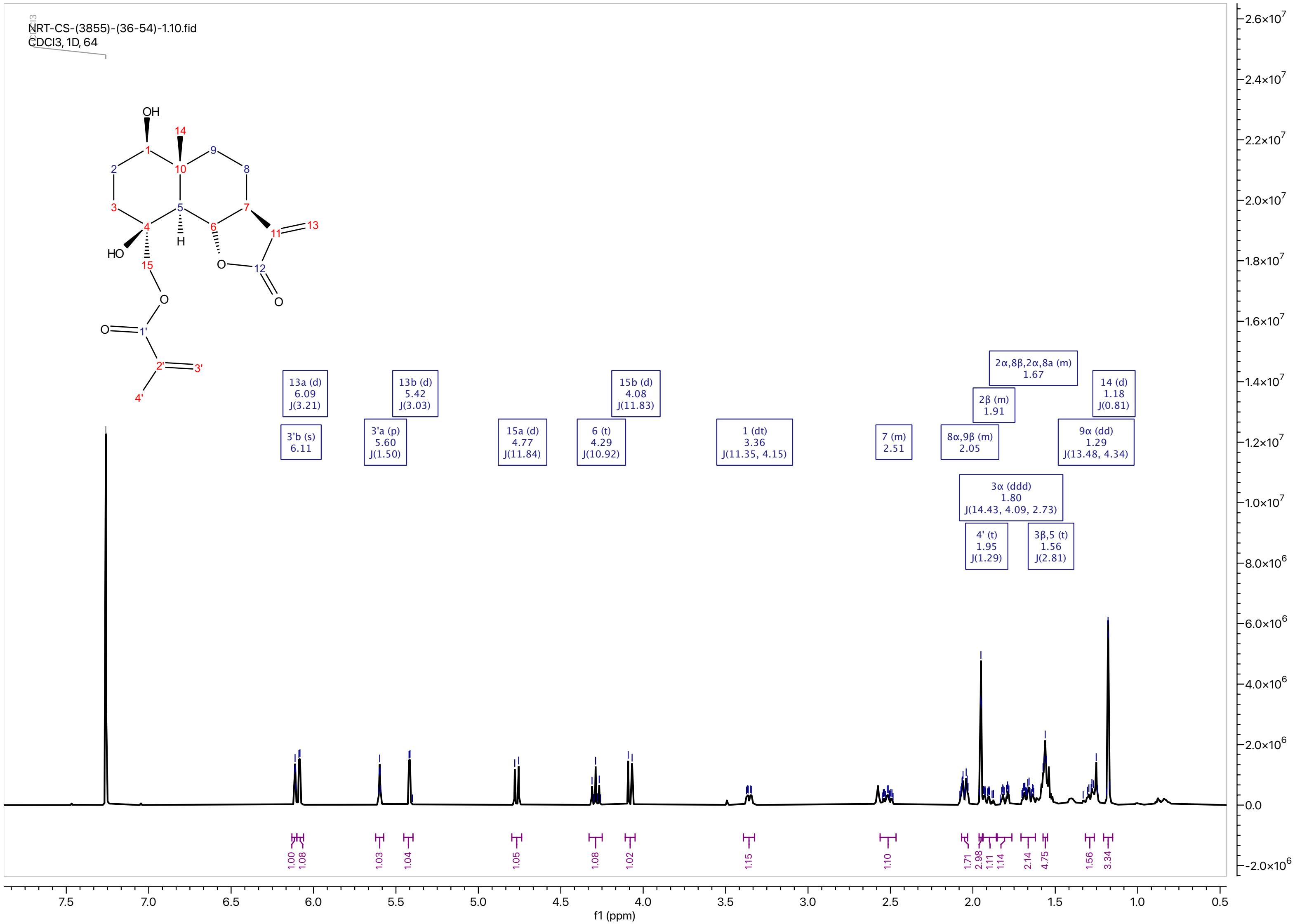


**Figure S2 :** SL2 (Vahlenin) $^1$H Proton 1D-NMR Spectrum (500 MHz, $CDCl_3$)

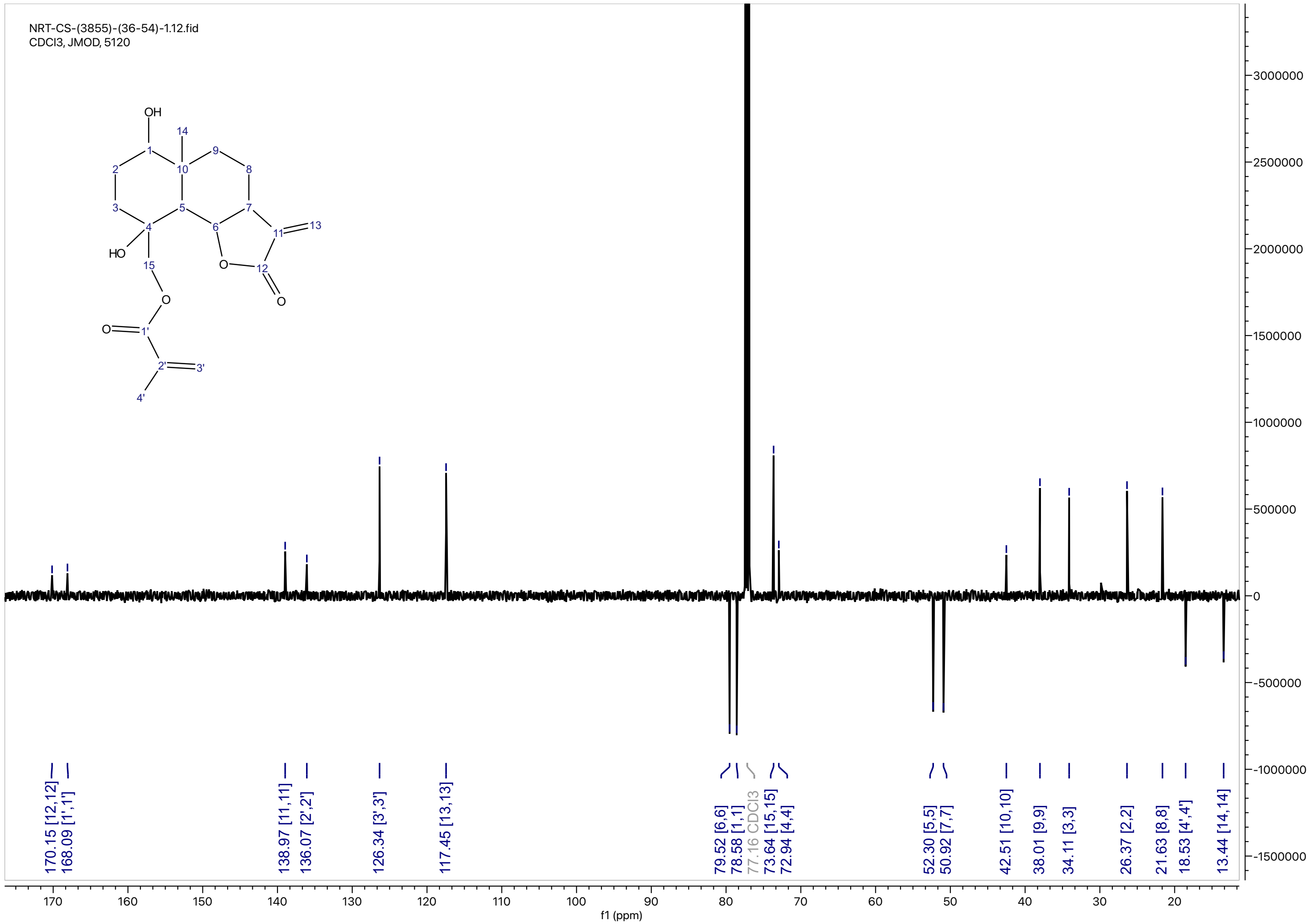


**Figure S3 :** SL2 (Vahlenin) $^{13}$C (APT) 1D-NMR Spectrum (125 MHz, $CDCl_3$)

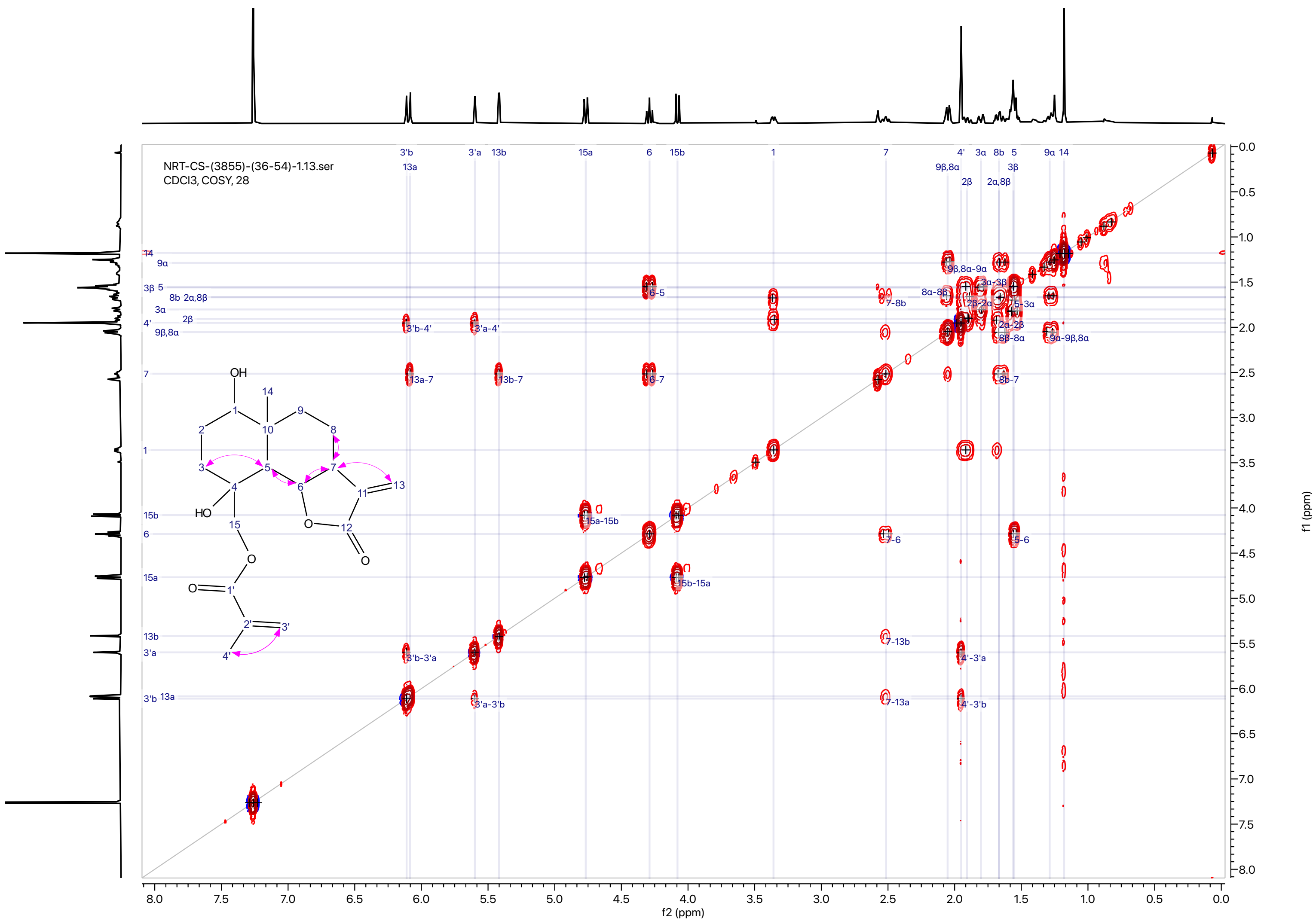


**Figure S4 :** SL2 (Vahlenin) COSY 2D-NMR Spectrum

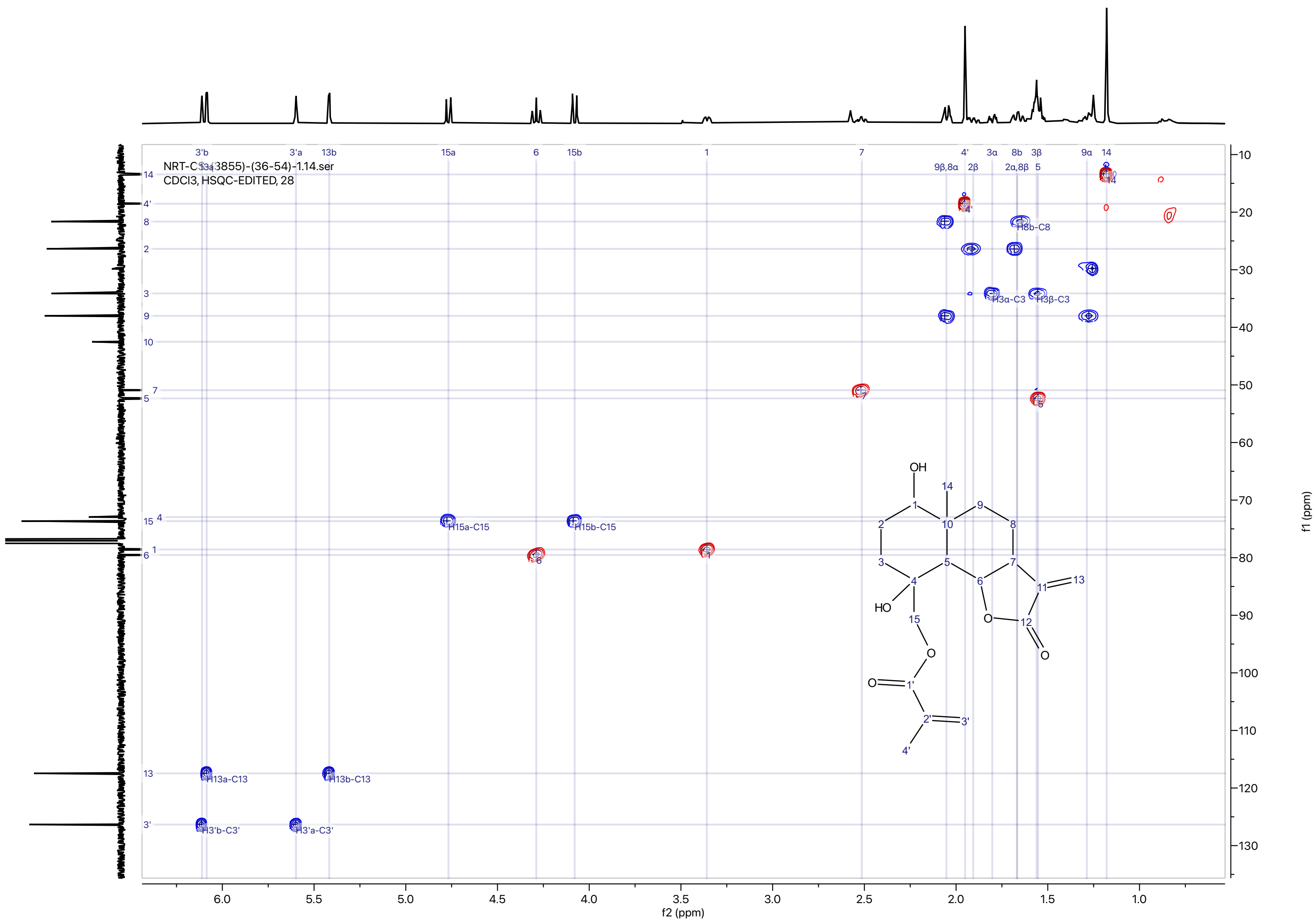


**Figure S5 :** SL2 (Vahlenin) HSQC 2D-NMR Spectrum

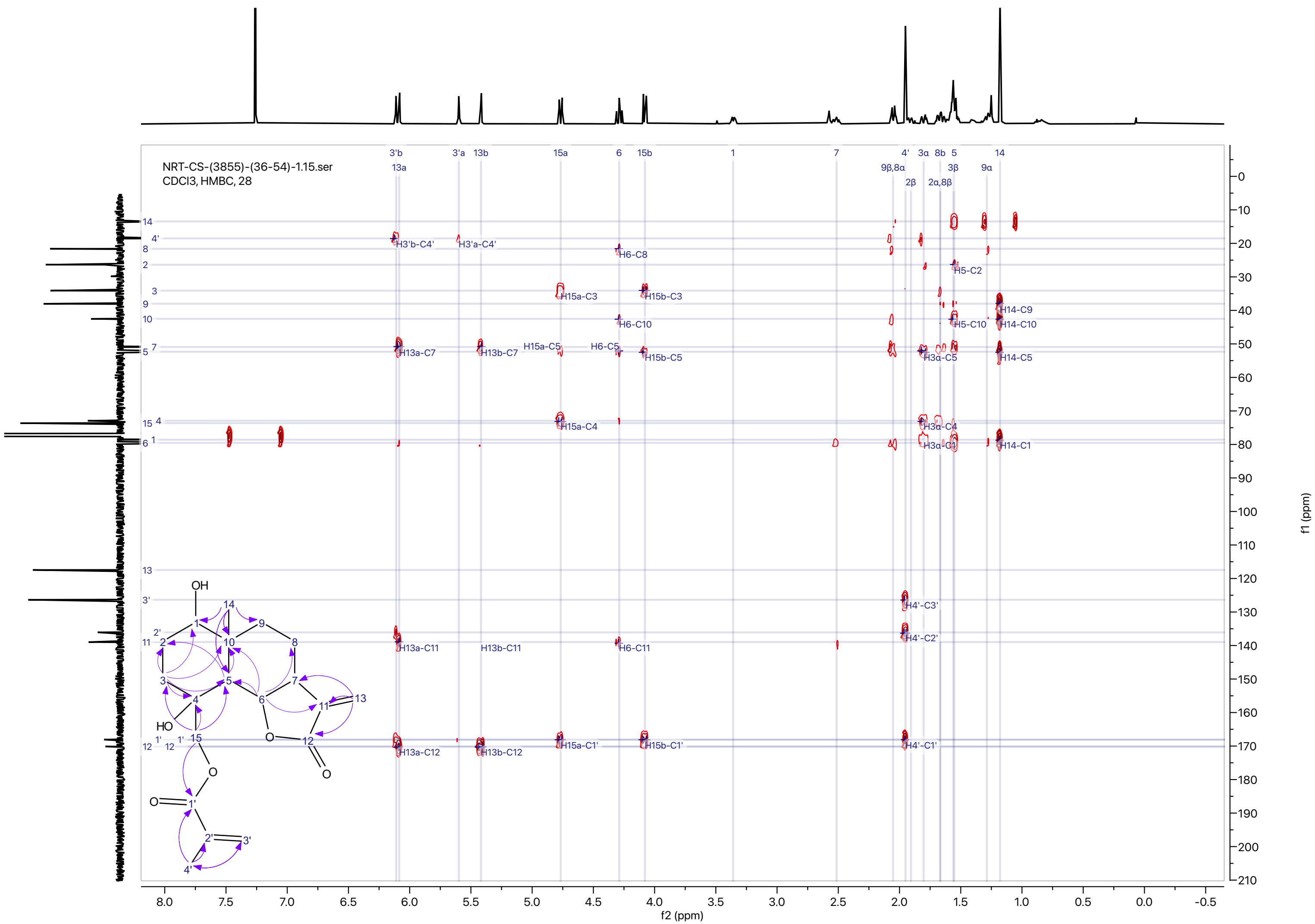


**Figure S6 :** SL2 (Vahlenin) HMBC 2D-NMR Spectrum

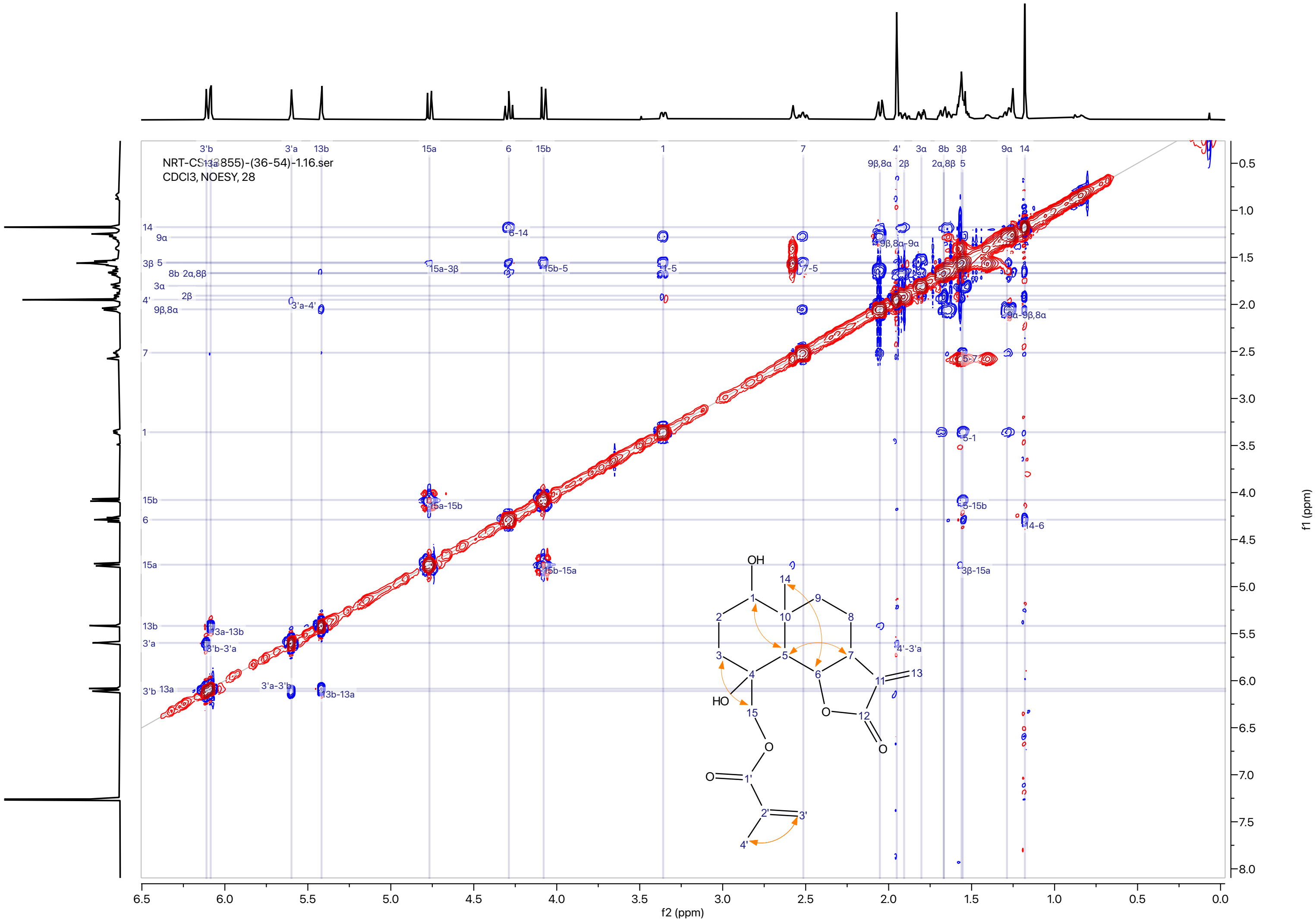

**Figure S7 :** SL2 (Vahlenin) NOESY 2D-NMR Spectrum

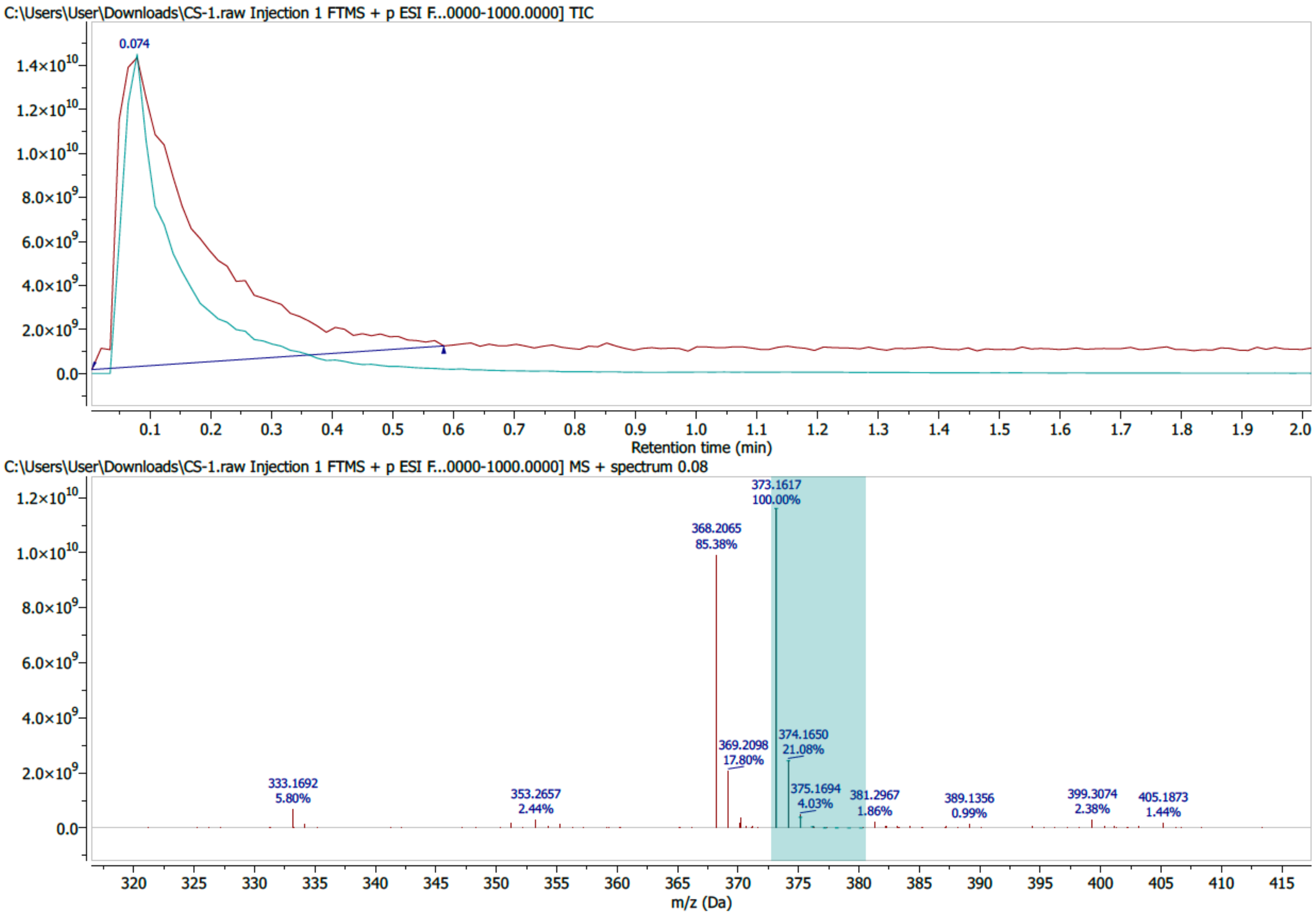


**Figure S8 :** SL2 (Vahlenin) Mass Spectrum (*m/z* 373.1617 $[M + Na]^+$ (calcd *m/z* 373.1622); error: -.499 ppm)

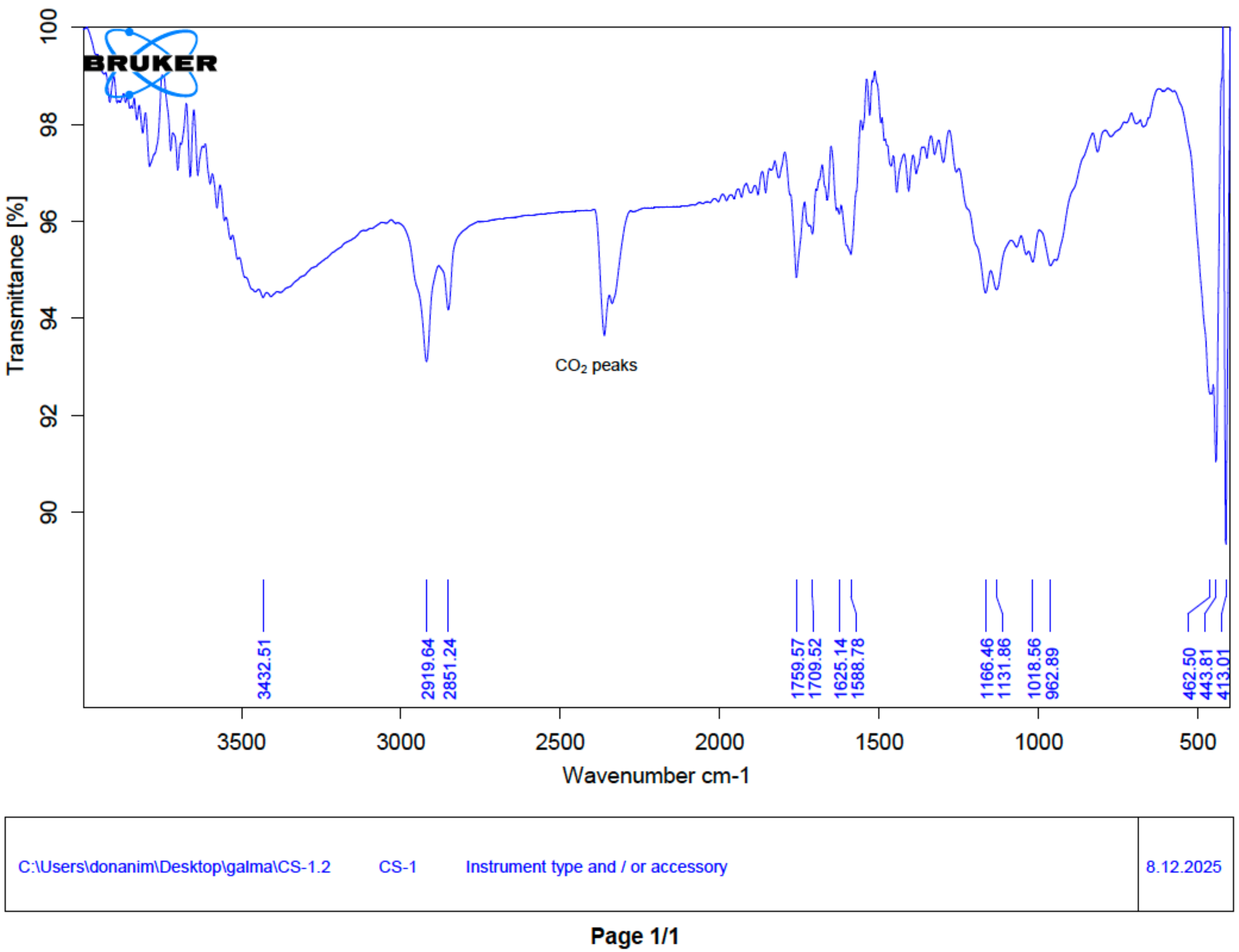


**Figure S9 :** SL2 (Vahlenin) IR Spectrum

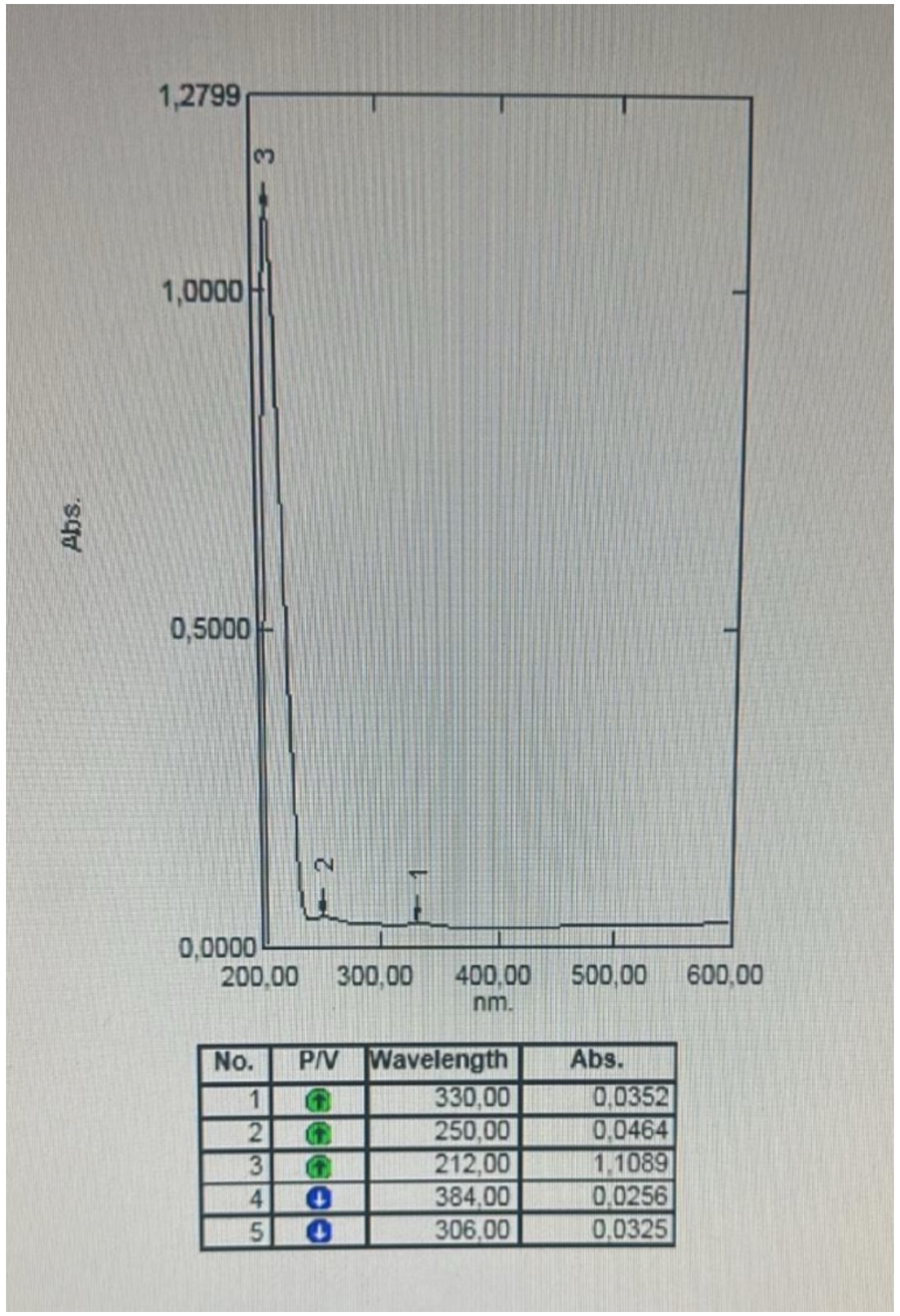


| No. | P/V | Wavelength | Abs. |
|---|---|---|---|
| 1 | ↑ | 330,00 | 0,0352 |
| 2 | ↑ | 250,00 | 0,0464 |
| 3 | ↑ | 212,00 | 1,1089 |
| 4 | ↓ | 384,00 | 0,0256 |
| 5 | ↓ | 306,00 | 0,0325 |

**Figure S10 :** SL2 (Vahlenin) UV spectrum (in methanol, 0.05 mg/mL)

**Table S2:** SL3 (Maximolide) HMBC, COSY, NOESY corelations

| No | HMBC | COSY | NOESY |
|---|---|---|---|
| 1 | - | H4(2.24), H5(2.24), H9β(2.24), H14b(4.76) | H5(2.24) |
| 2 | C1(39.7), C10(143.9) | - | - |
| 4 | - | H1(3.12), H15(1.24) | H6(3.93) |
| 5 | C8(76.0) | H1(3.12), H6(3.93), H15(1.24) | H1(3.12), H15(1.24) |
| 6 | C5(47.5) | H5(2.24), H7(2.24) | H4(2.24), H6(3.93), H8(3.76), H11(2.60) |
| 7 | C6(83.2), C8(76.0) | H6(3.93), H8(3.76), H9α(2.83), H11(2.60) | H13(1.44) |
| 8 | - | H7(2.24), H9α(2.83) | H6(3.93), H11(2.60) |
| 9α | C1(39.7), C7(54.1), C8(76.0), C10(143.9), C14b(115.1) | H7(2.24), H8(3.76), H9β(2.24) | H14a(5.06) |
| 9β | C8(76.0) | H1(3.12), H9α(2.83), H14a(5.06) | - |
| 10 | H2(2.50), H9α(2.83) | - | - |
| 11 | C12(178.6) | H7(2.24), H13(1.44) | H6(3.93), H8(3.76) |
| 12 | H11(2.60), H13(1.44) | - | - |
| 13 | C7(54.1), C12(178.6), C11(41.3) | H11(2.60) | H7(2.24) |
| 14a | C1(39.7), C9β(49.4) | H9β(2.24) | H9α(2.83) |
| 14b | C1(39.7), C9β(49.4) | H1(3.12) | - |
| 15 | - | H4(2.24), H5(2.24) | H5(2.24) |

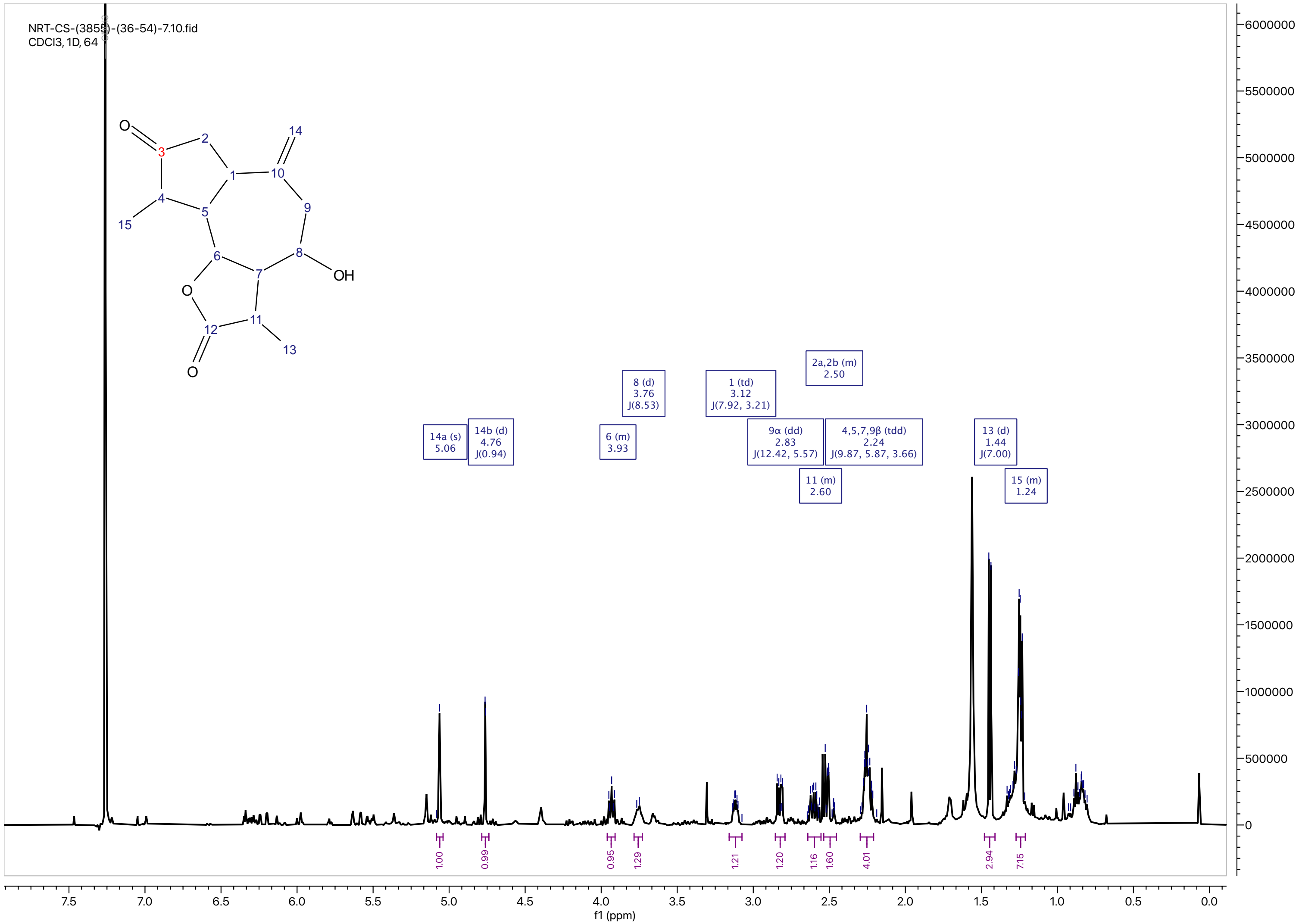


**Figure S11:** SL3 (Maximolide) 1D-$^{1}$H Proton NMR Spectrum (500 MHz, CDCl$_3$)

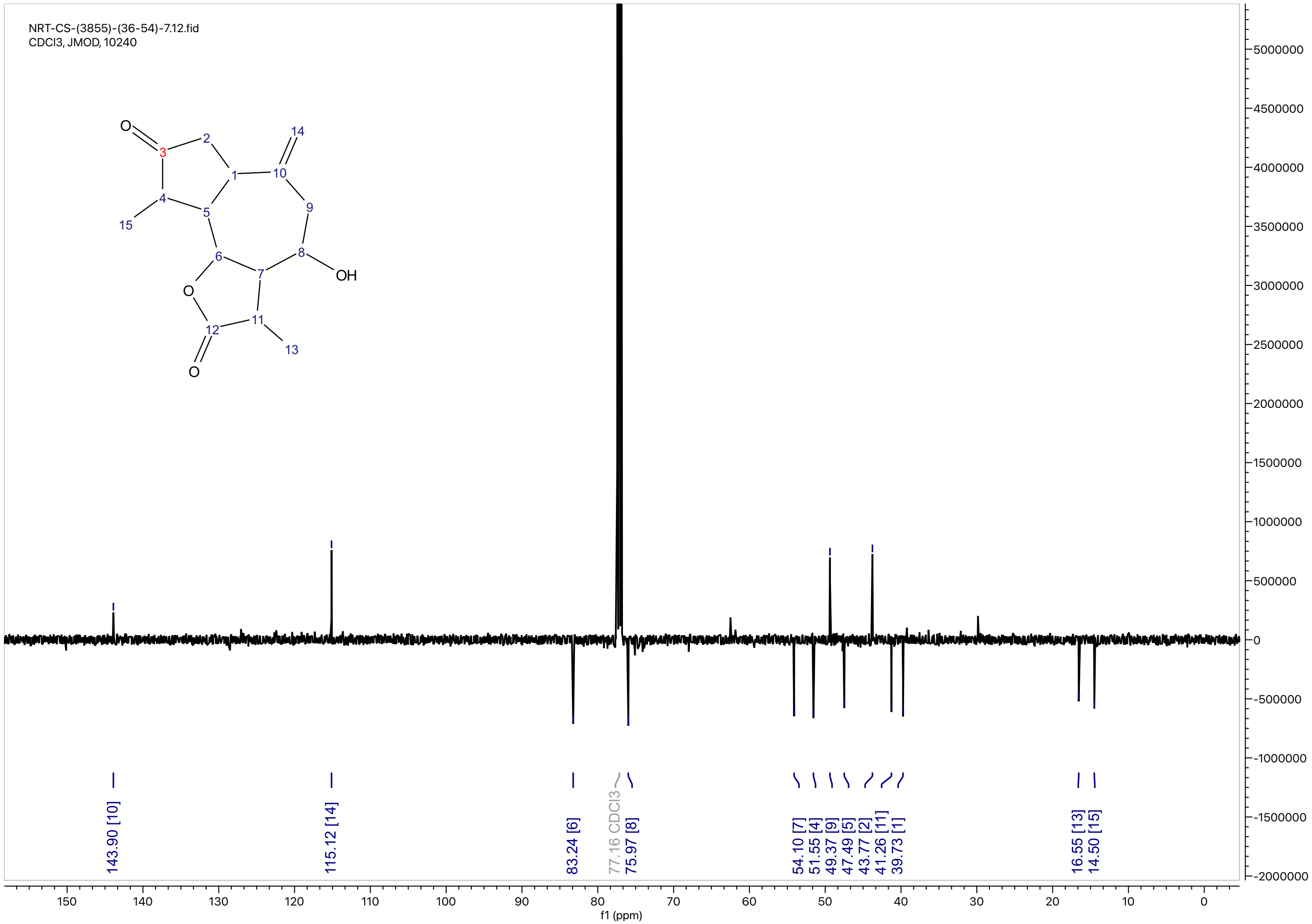


**Figure S12:** SL3 (Maximolide) $^{13}C$ (APT) 1D-NMR (125 MHz, $CDCl_3$)

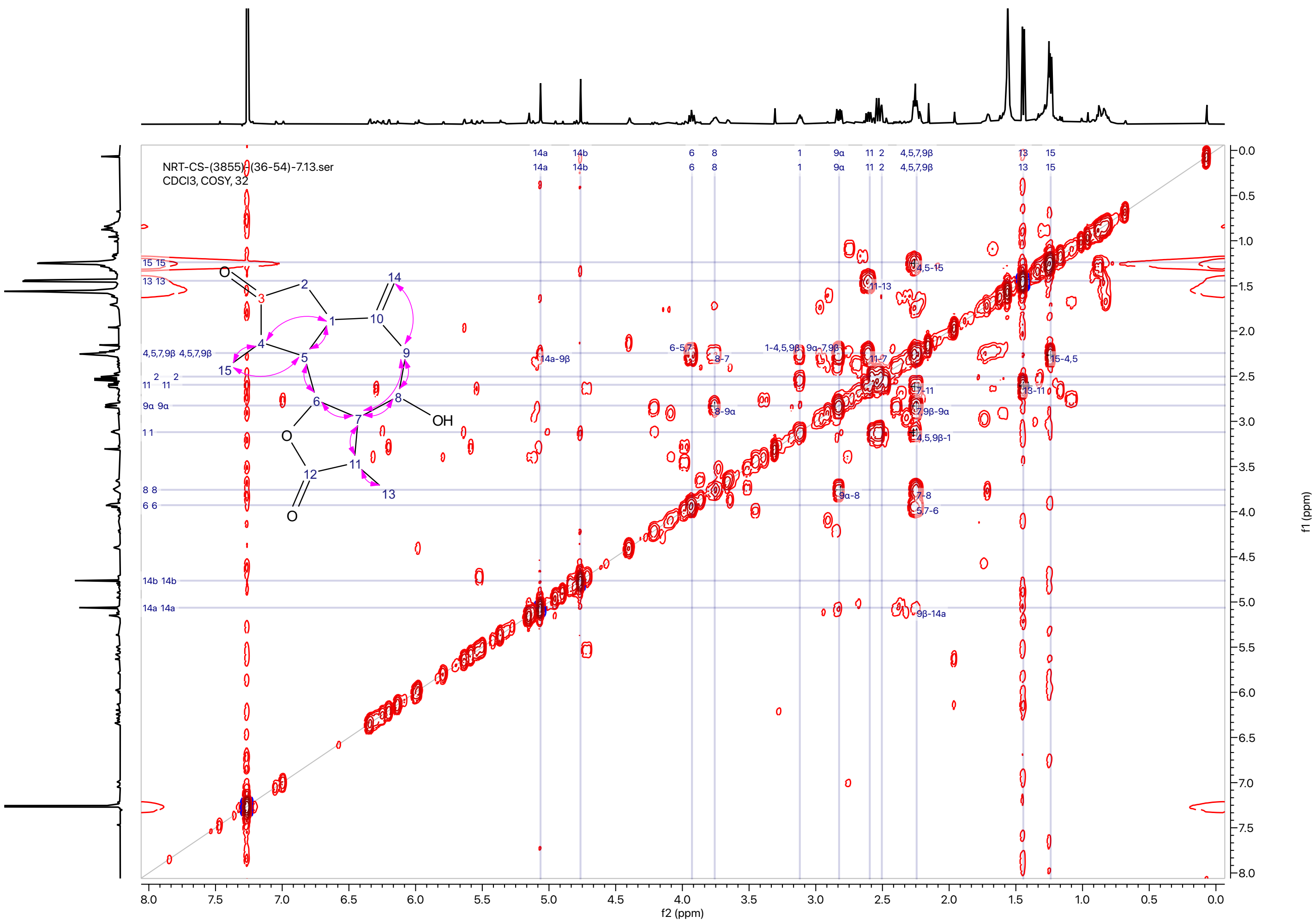

**Figure S13:** SL3 (Maximolide) COSY 2D-NMR Spectrum

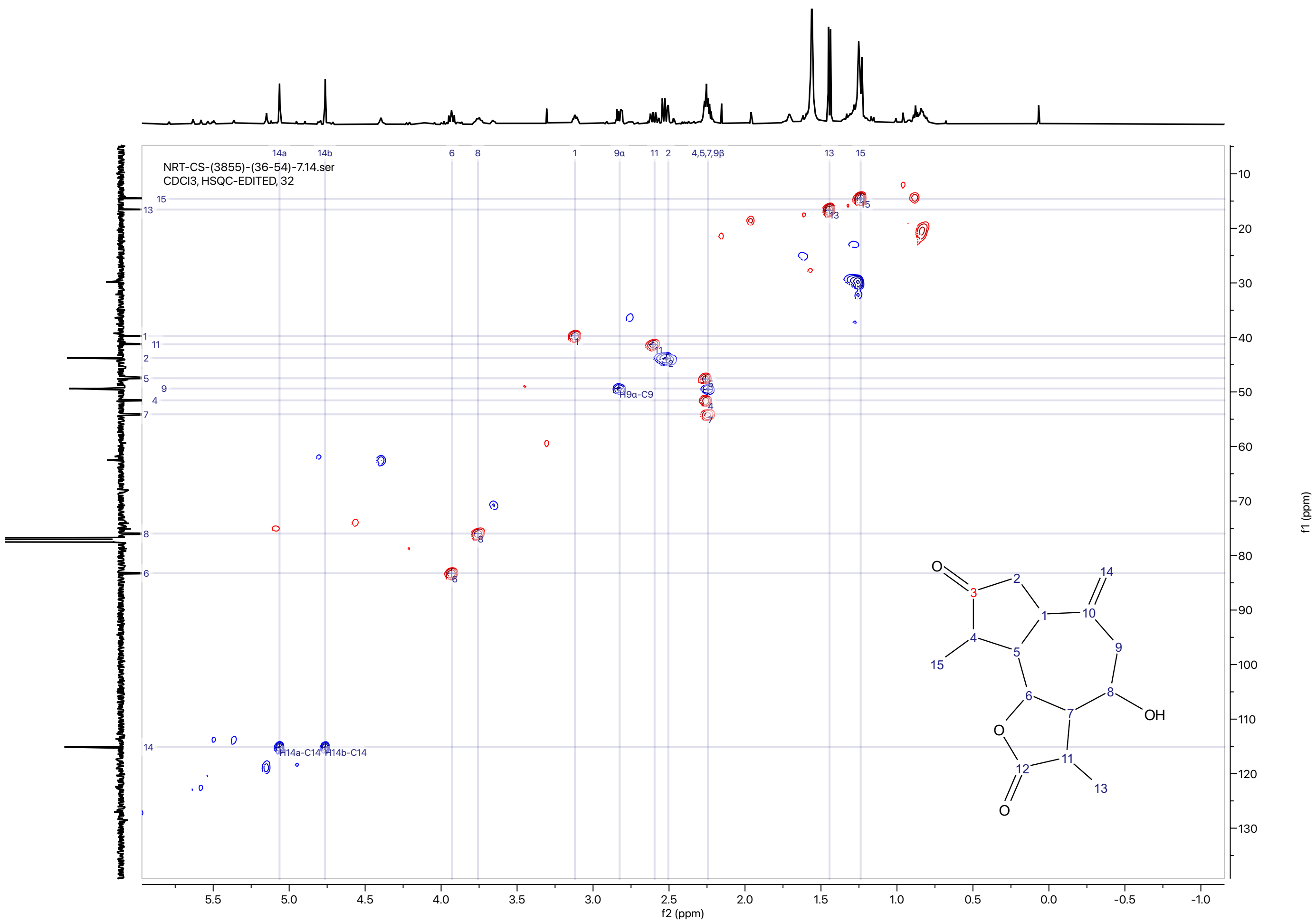


**Figure S14 :** SL3 (Maximolide) HSQC 2D-NMR Spectrum

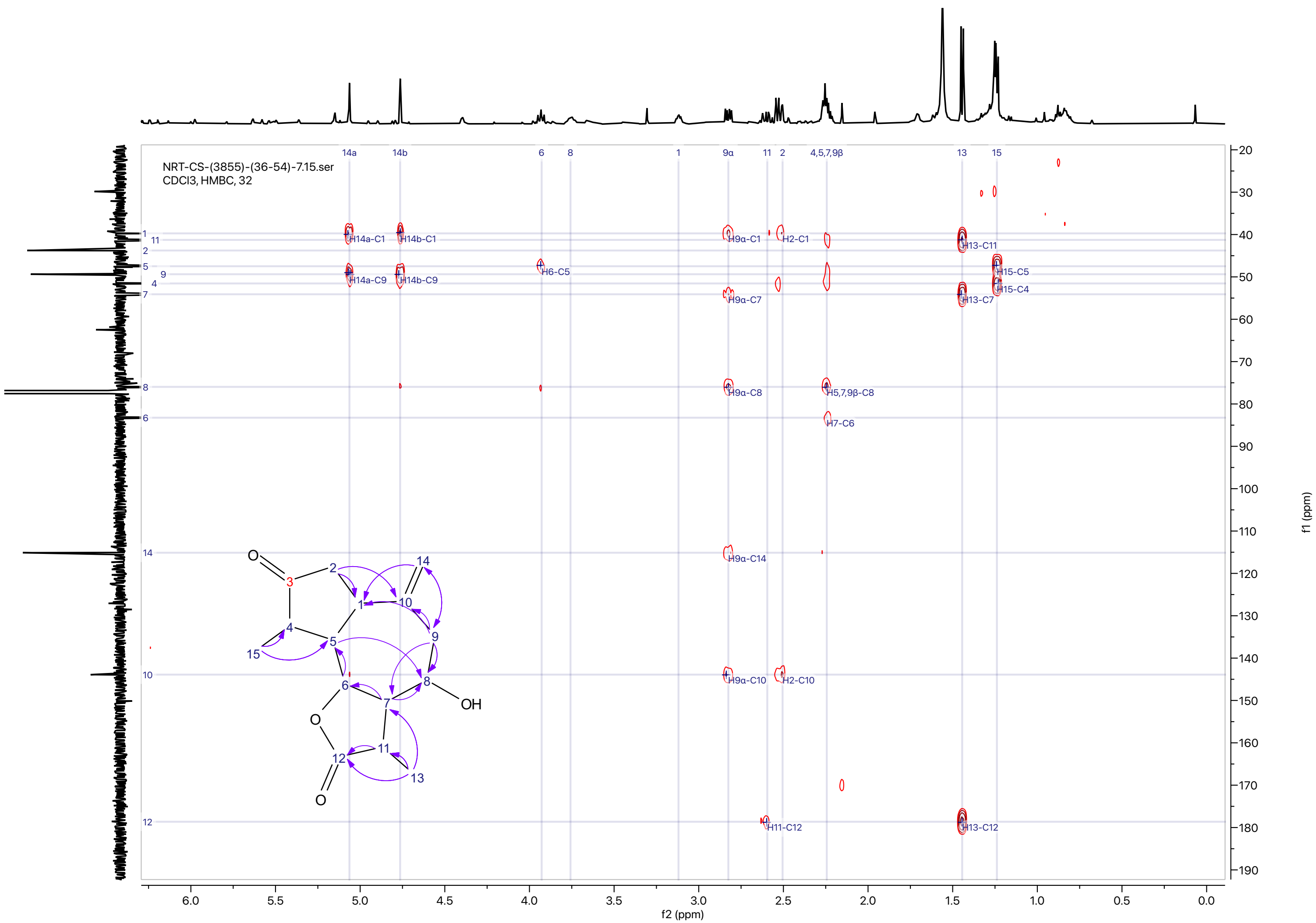


**Figure S15:** SL3 (Maximolide) HMBC 2D-NMR Spectrum

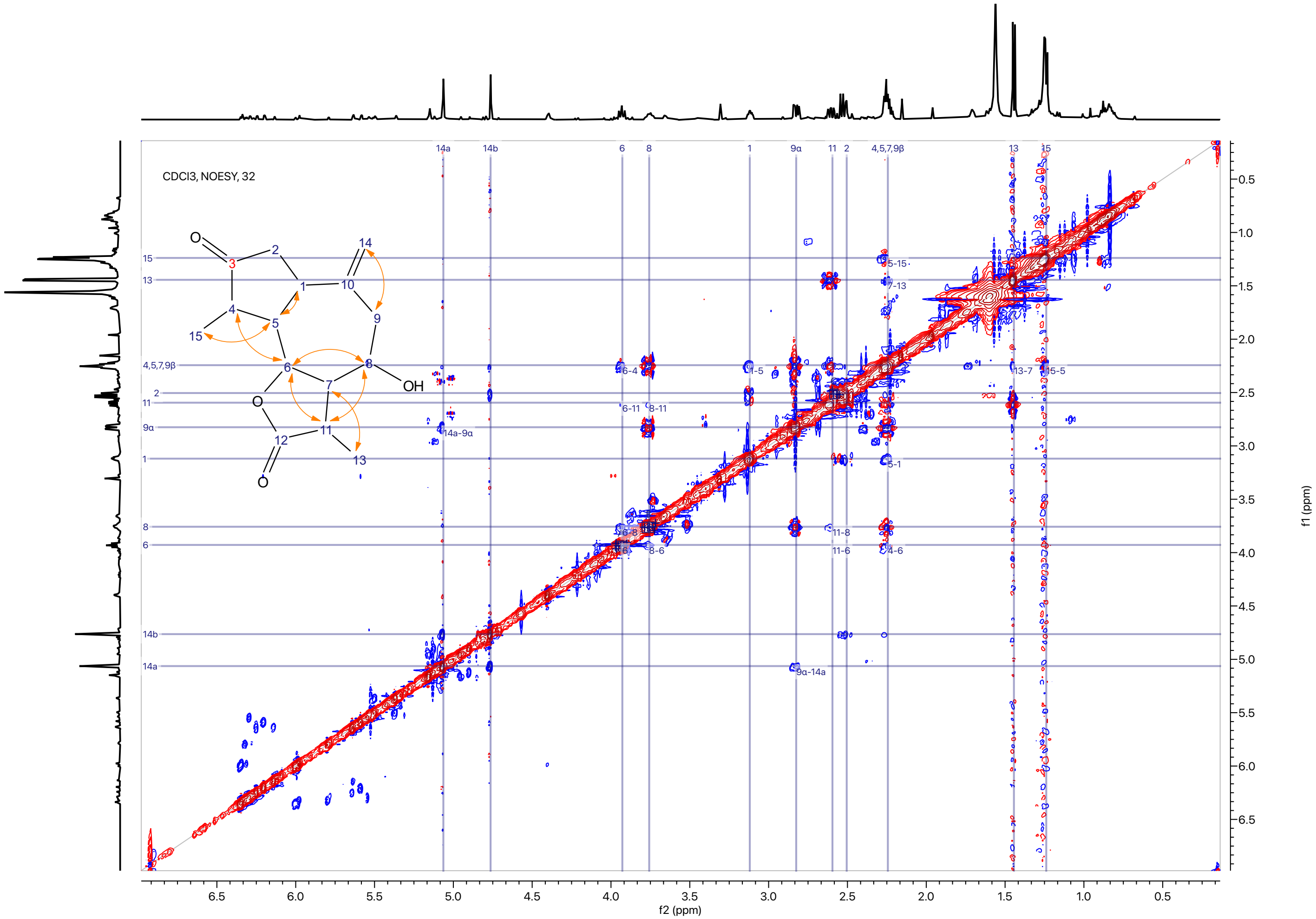


**Figure S16 :** SL3 (Maximolide) NOESY 2D-NMR Spectrum

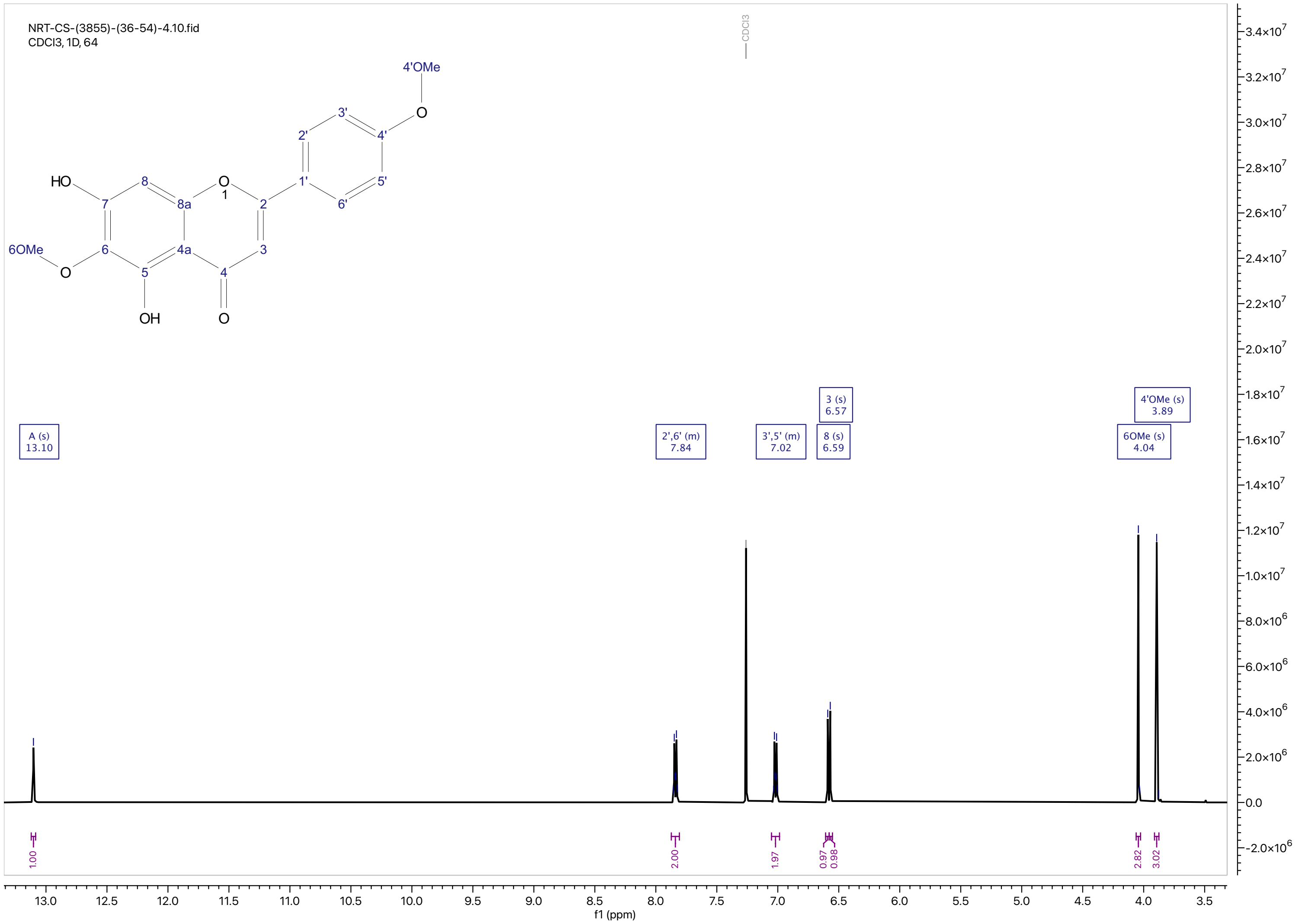


**Figure S17 :** F1 (Pectolinaringenin) 1D-[1]H Proton NMR Spectrum (500 MHz, $CDCl_3$)

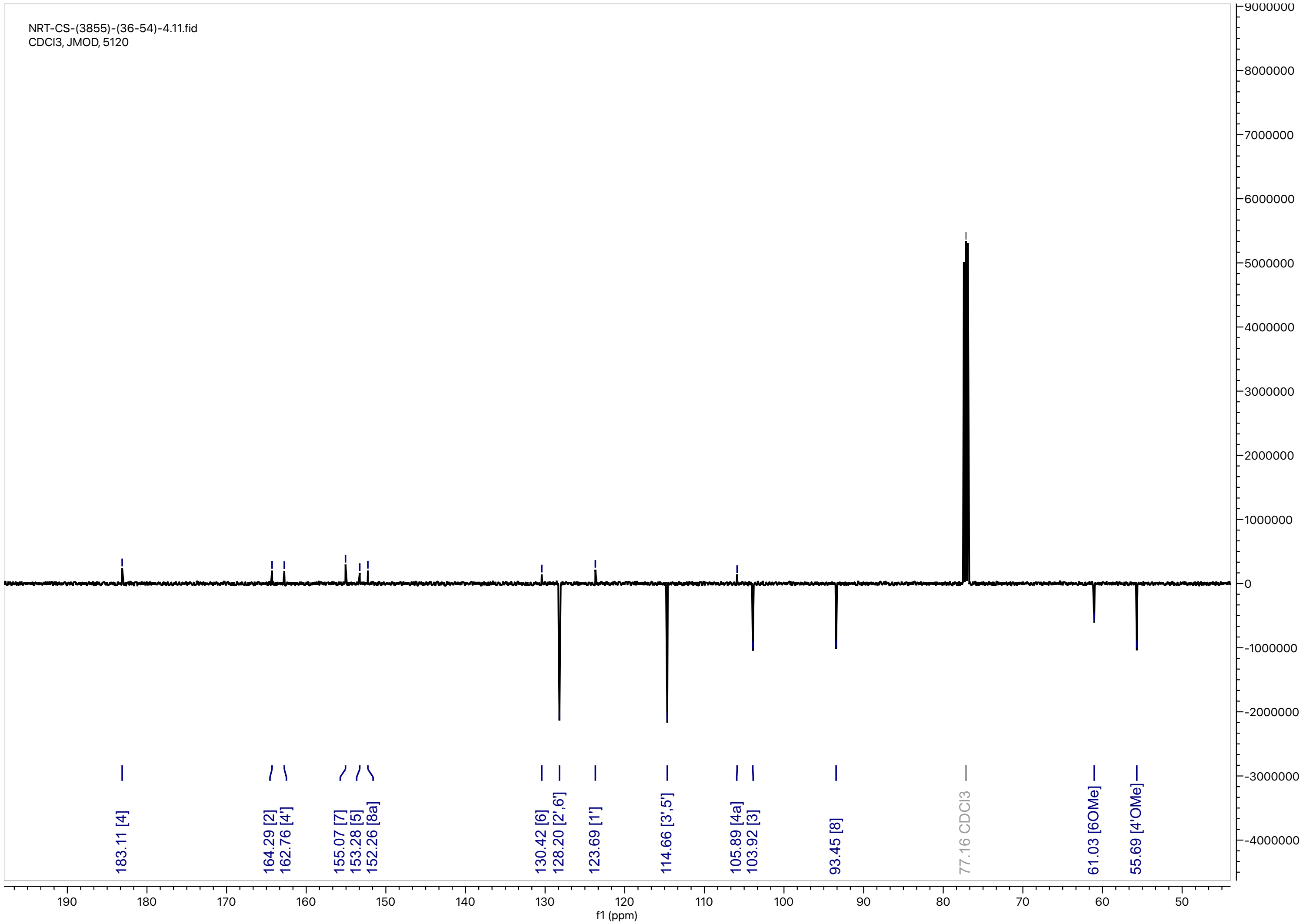


**Figure S18: F1** (Pectolinaringenin) $^{13}C$ (APT) 1D-NMR (125 MHz, $CDCl_3$)

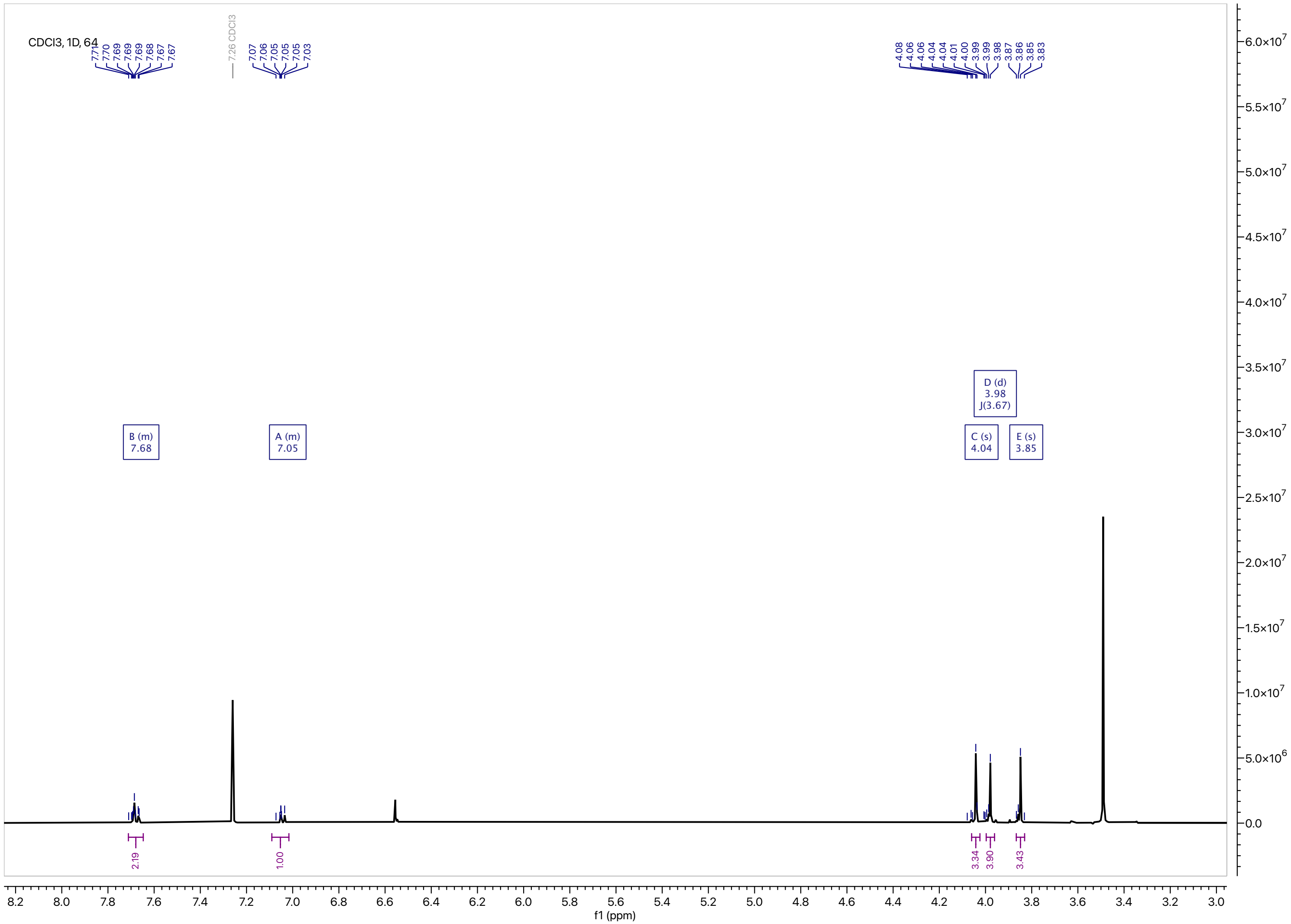


**Figure S19 :** F2 (Centaureidin) 1D-$^{1}$H Proton NMR Spectrum (500 MHz, $CDCl_3$)

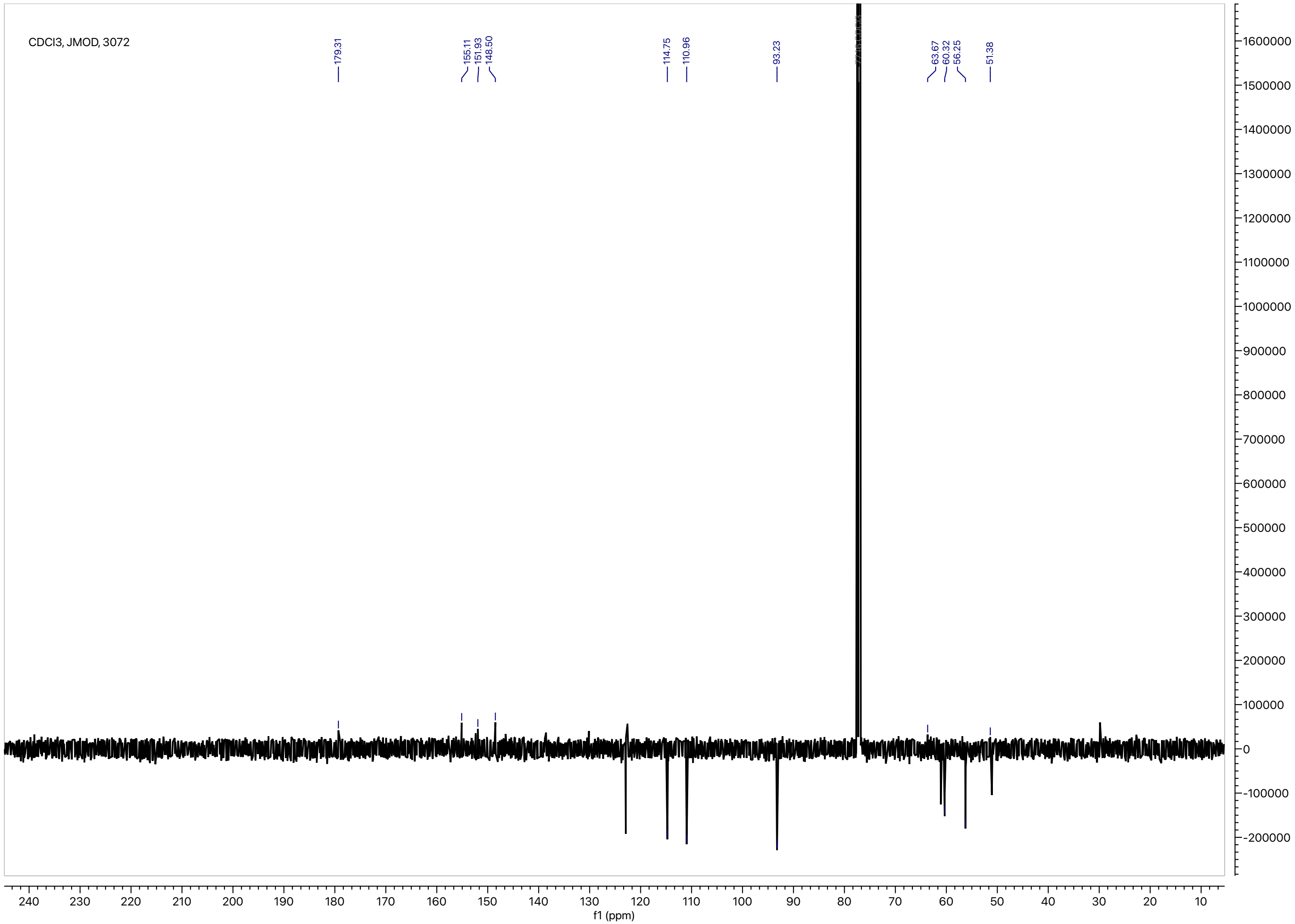


**Figure S 20 :** F2 (Centaureidin) $^{13}C$ (APT) 1D-NMR (125 MHz, $CDCl_3$)

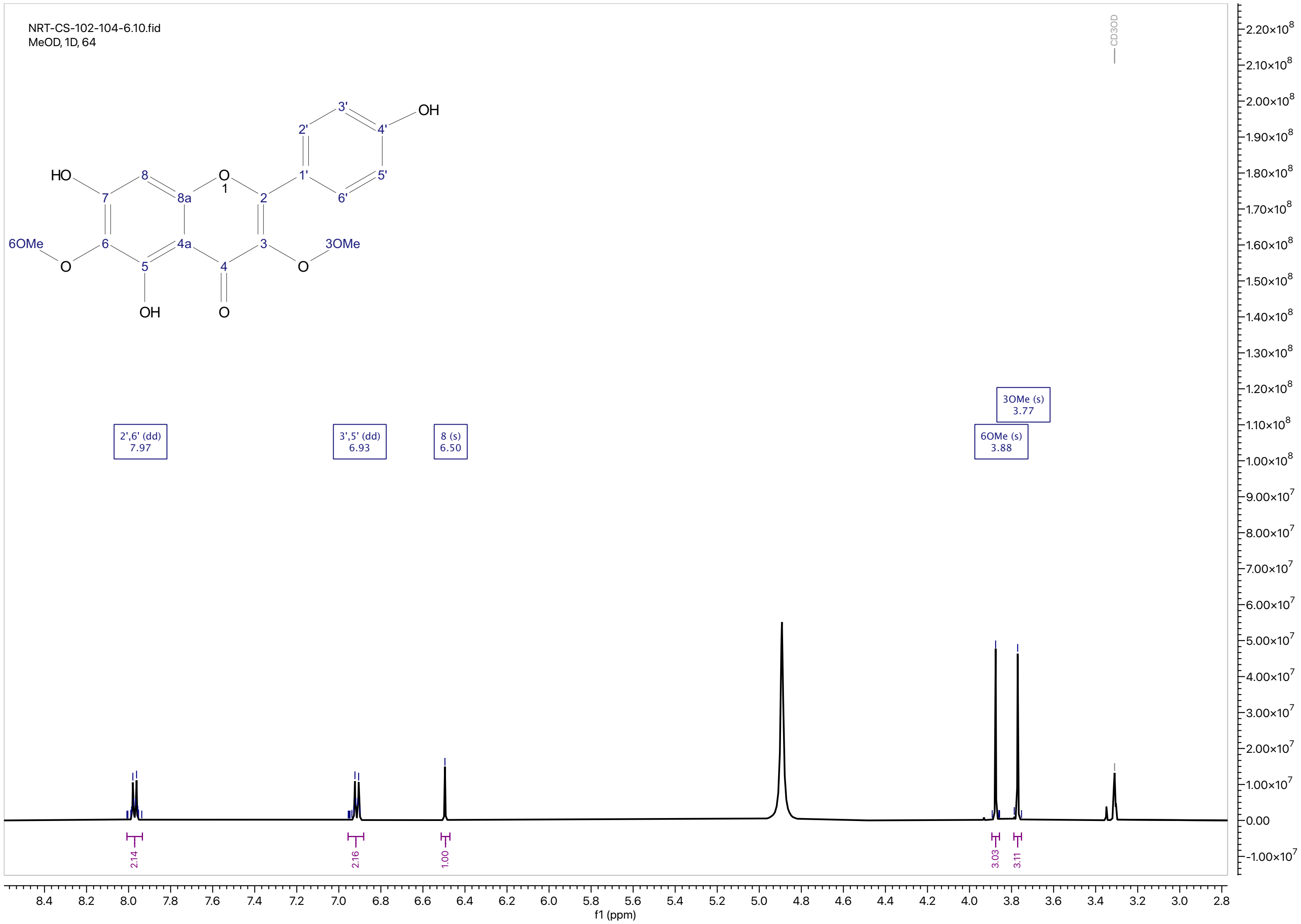


**Figure S21:** F3 (Axillarin) 1D-$^{1}$H Proton NMR Spectrum (500 MHz, MeOD)

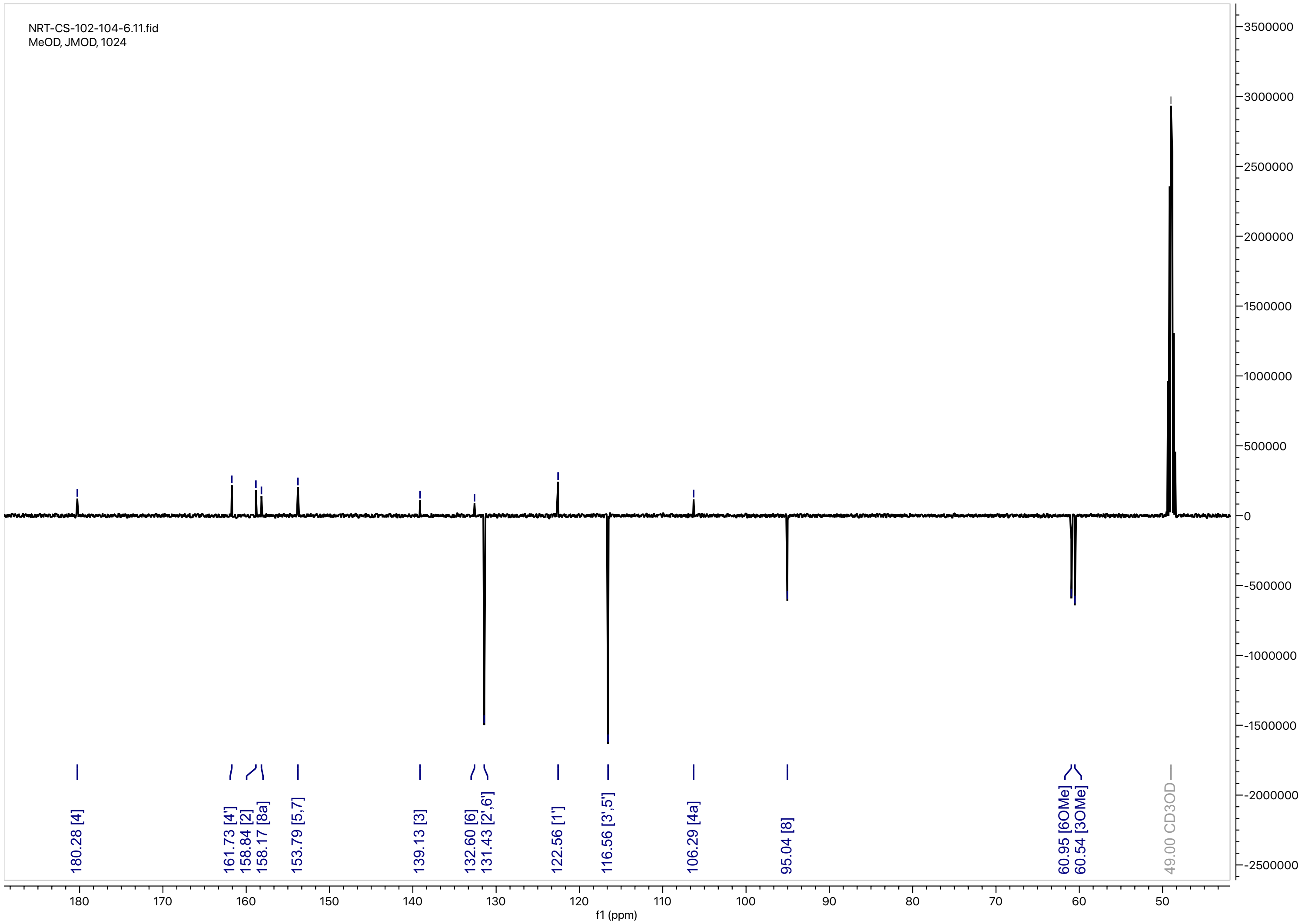


**Figure S22:** F3 (Axillarin) $^{13}$C (APT) 1D-NMR (125 MHz, MeOD)
